# Tumour immune characterisation of primary triple-negative breast cancer using automated image quantification of immunohistochemistry-stained immune cells

**DOI:** 10.1038/s41598-024-72306-1

**Published:** 2024-09-13

**Authors:** Suze Roostee, Daniel Ehinger, Mats Jönsson, Bengt Phung, Göran Jönsson, Gottfrid Sjödahl, Johan Staaf, Mattias Aine

**Affiliations:** 1 Division of Oncology, Department of Clinical Sciences Lund, Lund University, Medicon Village, 22381 Lund, Sweden; 2 Division of Translational Cancer Research, Department of Laboratory Medicine, Lund University, Medicon Village, 22381 Lund, Sweden; 3 Department of Genetics, Pathology, and Molecular Diagnostics, Skåne University Hospital, Lund, Sweden; 4 Department of Translational Medicine, Lund University, Malmö, Sweden

**Keywords:** Breast cancer, Biomarkers, Cancer genomics

## Abstract

The tumour immune microenvironment (TIME) in breast cancer is acknowledged with an increasing role in treatment response and prognosis. With a growing number of immune markers analysed, digital image analysis may facilitate broader TIME understanding, even in single-plex IHC data. To facilitate analyses of the latter an open-source image analysis pipeline, Tissue microarray MArker Quantification (TMArQ), was developed and applied to single-plex stainings for p53, CD3, CD4, CD8, CD20, CD68, FOXP3, and PD-L1 (SP142 antibody) in a 218-patient triple negative breast cancer (TNBC) cohort with complementary pathology scorings, clinicopathological, whole genome sequencing, and RNA-sequencing data. TMArQ’s cell counts for analysed immune markers were on par with results from alternative methods and consistent with both estimates from human pathology review, different quantifications and classifications derived from RNA-sequencing as well as known prognostic patterns of immune response in TNBC. The digital cell counts demonstrated how immune markers are coexpressed in the TIME when considering TNBC molecular subtypes and DNA repair deficiency, and how combination of immune status with DNA repair deficiency status can improve the prognostic stratification in chemotherapy treated patients. These results underscore the value and potential of integrating TIME and specific tumour intrinsic alterations/phenotypes for the molecular understanding of TNBC.

## Introduction

Breast cancer is the most common malignancy in women worldwide^1^. Triple-negative breast cancer (TNBC), a subtype that lacks the expression of the oestrogen receptor, progesterone receptor and amplification of the *ERBB2*/*HER2* gene, is a particularly aggressive form that presents a unique clinical challenge. TNBC patients often face poor outcomes due to the lack of targeted therapies^2^.

TNBC accounts for about 10% of all breast cancer cases, often affecting younger individuals and leading to early relapses. TNBC tumours are characterised by highly rearranged genomes and closely linked to mutations in high-risk breast cancer genes like *BRCA1* and *BRCA2.* The latter links TNBC with DNA repair deficiency, such as homologous recombination deficiency (HRD), which has been associated with improved prognosis after adjuvant chemotherapy^3,4^. Recent research has highlighted the importance of the immune response in determining the outcomes of early-stage TNBC patients, regardless of whether they receive chemotherapy^5–7^. Here, the infiltration of different immune cell types in the tumour immune microenvironment (TIME) may represent the systemic anticancer immune response towards tumour characteristics that are yet not fully understood. The composition and density of different immune cell populations in the TIME likely influence tumour progression and success of anti-cancer therapies profoundly, although our understanding of these processes is still incomplete. Different methods exist to measure the immune response in tumour tissue samples, including the morphological assessment of the level of stromal tumour infiltrating lymphocytes (sTILs) on H&E-slides^5^, IHC-staining of cell type specific markers, immune cell type directed flow cytometry, or genomic methods such as mRNA expression analyses^8^. Within TNBC exists a large heterogeneity in the level of immune response across individual tumours^9,10^. This immune heterogeneity has also been recognized in proposed transcriptional TNBC subtypes through an immunomodulatory (IM) subtype/phenotype reported in 2011 by Lehmann et al.^11^. While the IM subtype was considered as one of six intrinsic TNBC subtypes, this subtyping scheme was later refined to comprise four subtypes (BL1, BL2, M, LAR) where the IM subtype can be superimposed on the former as an independent feature of the tumour^12^.

To better understand interactions between somatic tumour alterations and the TIME, improvements in methods for the characterization of the TIME cell composition and cell type interactions are needed. Ideally, this involves both a quantitative identification of cell types using e.g., cell type specific markers, but also the ability to target the interrelationship between different markers expressed both in malignant and non-malignant cells based on their spatial organisation. Technological advancements have made it possible to analyse multiple markers simultaneously in a single FFPE section, but also through flow cytometry, mass spectrometry, or even more complex spatial profiling methods based on nucleic acid or protein detection^8,13^. While these technologies provide improved resolution and multiplex capability, they are often costly, can have specific tissue requirements, and have a limited throughput. As a result, single-marker immunohistochemistry (IHC) on FFPE whole slide sections or tissue microarray (TMA) sections remain the standard in routine breast cancer diagnostics and research. This means a wealth of archival slides and scanned image data is available for retrospective analysis, while many cutting-edge technologies rely on fresh sectioning, and analysis pipelines built around these are often not amenable to archival image data.

While throughput of both single-plex and multiplex methods are increasing, data analysis remains a bottleneck, especially when human interpretation or grading is needed. Despite standardisation efforts, human interpretation often requires subjective assessments to be made which in turn introduce bias^14,15^. To address this limitation digital image analysis tools have been developed, some incorporating deep-learning for different classification and scoring applications such as TIL-estimation^16,17^ and PD-L1 scoring^18^. Yet, challenges remain, such as accurately identifying individual cells and transferability of trained deep learning (DL) models to other IHC data. For the former, advancements in convolutional neural networks have improved the extraction of cellular metrics that are essential for definition of the spatial tissue architecture, like the shape, size, position, and count of individual cells^19^.

To facilitate TIME analysis an open-source digital image analysis pipeline, Tissue microarray MArker Quantification (TMArQ), was assembled that can quantitatively and spatially analyse single marker IHC images with high-throughput and reproducibility. To target TIME interactions with specific tumour phenotypes and alterations in TNBC we applied TMArQ to a large TMA data set comprising over 200 TNBCs from a molecularly very well-profiled, population-representative, TNBC cohort of patients^3^ that were IHC stained for multiple immune markers. As proof of concept, we also extended our analysis to one bladder cancer and one malignant melanoma TMA cohort. Through comparisons with matched genomic data as well as pathologist and DL scoring, we demonstrate that TMArQ generates a deterministic quantification of the TIME in TNBC and is on par with gold standard and more user intensive methods. In TNBC, TMArQ cell counts demonstrates how immune markers are coexpressed in the TIME when considering proposed molecular subtypes and DNA repair deficiency, that gene expression-based deconvolution methods should be interpreted with care, and how combination of immune status with DNA repair deficiency status can further improve the prognostic performance in adjuvant treated patients. These results underscore the value and potential of integrating TIME and specific tumour intrinsic alterations/phenotypes for enhancing our understanding of the interplay between genotype and phenotype in TNBC.

## Results

### Comparison of automated TMArQ pipeline cell counts to available pathology scores

**Fig. 1:**
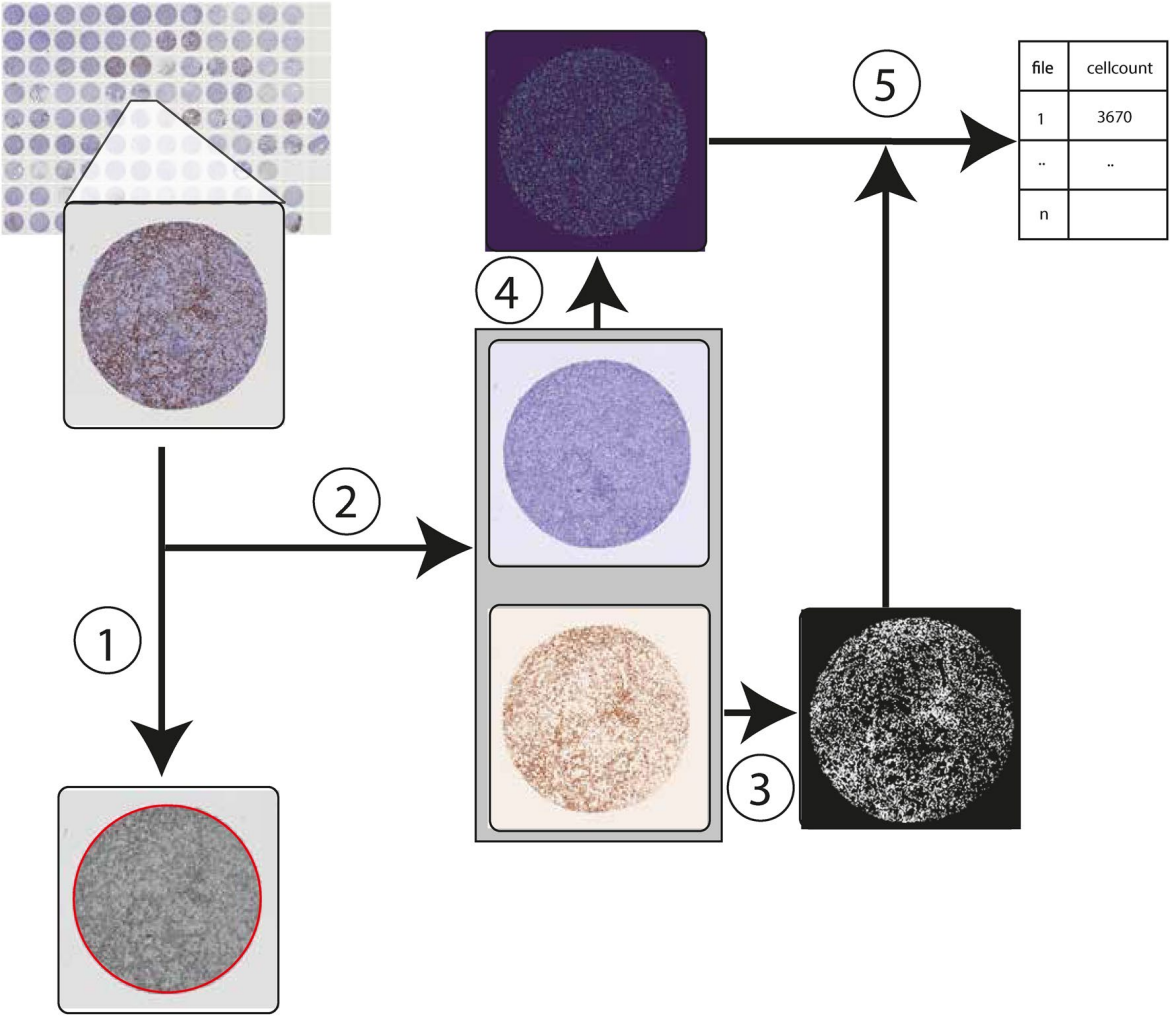
TMArQ Image analysis pipeline workflow. Outline of the TMArQ analysis approach based on the *starDist* cell segmentation algorithm. (1) Detection of region of interest (ROI) using Hough circle detection (red circle). (2) Colour deconvolution to separate out the DAB staining from the hematoxylin staining. (3) Thresholding signal in the DAB layer to determine DAB absence/presence on the level of individual pixels. (4) Cell nuclei segmentation using *starDist*. (5) Combining *starDist* detected cells with the DAB staining layer to count IHC-stained cells in the core.

TMArQ is an automated digital analysis pipeline based on open-source software as outlined in Fig. 1. To assess the pipeline’s performance and validity we applied it for automated IHC scoring of p53 and six immune cell antibodies as well as PD-L1 (SP142 antibody) in a large TNBC TMA cohort (n = 218) (Table 1, Supplementary Table 1, Supplementary Figs. 1 and 2). Following the application of the pipeline, we first compared the extracted log2 transformed cell counts to different marker score estimates made by breast cancer pathologists as outlined in our previous study^10^. This included scores for PD-L1 and CD20 (B-cell marker) on TMA core level (same stainings as for the automated pipeline), and for TIL percentages on whole-slide H&E-stained tumour sections from the same FFPE tissue blocks that were used for TMA construction.

**Table 1:** Sample and patient characteristics for the SCAN-B TNBC cohort.

	Samples	Unique samples
TMA marker		
CD3	436	218
CD4	436	218
CD8	436	218
CD20	436	218
CD68	436	218
PD-L1 (SP142)	436	218
FOXP3	436	218
p53	436	218
TMA block		
1	104	52
2	96	48
3	96	48
4	50	25
5	90	45
Grade		
2	50	25
3	376	188
NA	10	5
HRD status		
High	256	128
Low/inter	176	88
NA	4	2
TNBCtype (Lehmann-4)		
BL1	158	79
BL2	88	44
LAR	78	39
M	104	52
NA	8	4
Lymph node status		
Negative	270	135
Positive	162	81
NA	4	2
PAM50 subtype		
Basal	344	172
Her2	64	32
LumA	8	4
LumB	2	1
Normal	12	6
unclassified	2	1
NA	4	2

**Fig. 2:**
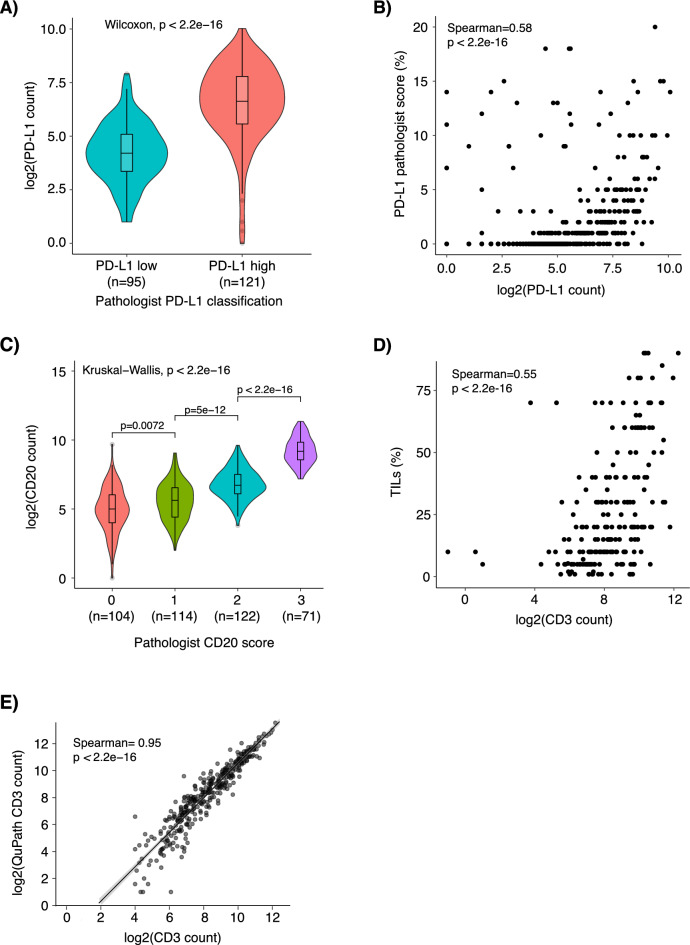
Comparison of TMArQ cell counts to pathologist scoring and QuPath scores in TNBC. (**A**) Violin plots showing the distribution of TMArQ cell counts of PD-L1 (SP142) to pathologist scores of PD-L1 low (< 1% IC) or PD-L1 high (≥ 1% IC). (**B**) Scatterplot showing the correlation of PD-L1 pipeline counts for each core to corresponding pathology estimated SP142 PD-L1 scores in %. (**C**) Violin plots showing the distribution of CD20 pipeline counts to binned pathologist CD20 scores. (**D**) Scatterplot showing the correlation of CD3 pipeline counts from TMA cores to pathology TIL percentages estimated from matched H&E-stained whole slide sections. (**E**) Scatterplot showing the comparison of TMArQ core CD3 counts to QuPath CD3 core counts. Log transformation of TMArQ counts was performed before analyses.

To investigate whether our automated pipeline scores could be used to capture a clinically relevant antibody cut-off, we compared automated PD-L1 cell counts using the Roche SP142 antibody to the established clinical cut-off of 1% immune cell (IC) staining for the antibody. Figure 2A shows a clear separation (albeit a degree of overlap) in number of PD-L1 stained cells from the pipeline for individual cores versus the binned pathology groups of PD-L1-low (< 1% IC) or PD-L1-high (≥ 1% IC), demonstrating that the pipeline counts are consistent with pathologist scoring. We also calculated the correlation of the PD-L1 pipeline cell counts to pathologist estimated PD-L1 percentage scores (Fig. 2B). Here we also observed a trend of increasing pipeline counts corresponding to higher PD-L1 pathology scores with a Spearman correlation of 0.58.

To evaluate our automated pipeline against human-derived scores on a semi-quantitative (ordinal) scale, we compared the automated pipeline counts to available pathology scores for the B-cell marker CD20. For this analysis CD20 antibody presence was previously scored using a four-level scale by a pathologist, where 0 corresponds to no/lowest CD20 staining^10^. Figure 2C shows that higher pipeline cell counts correspond to increasing pathology scores. In particular, the increase in corresponding pipeline counts when moving from a score of 2 to 3 is notable.

Finally, we compared pathology-estimated TIL proportions (%) to our automated CD3 (a pan-T-cell marker) cell counts. For every tumour we compared the mean CD3 count of the two cores to the estimated TIL percentage. We found a Spearman correlation of 0.55 between an increased CD3 + cell count in the TMA cores and an increased TIL percentage from the whole slide analysis (Fig. 2D). Taken together, these comparisons support the notion that the output from of our digital pipeline is in good agreement with independently derived estimates by a human evaluator.

### Comparison of TMArQ results for CD3 to corresponding QuPath scores

We compared TMArQ against QuPath^20^, a software for image analysis of histological slides, using a custom script for QuPath to automate the image processing. Comparison of CD3 + computed cell counts in individual cores from both methods demonstrated a high agreement between methods (Spearman correlation = 0.95, Fig. 2E).

### Comparison of TMArQ automated pipeline results to matched bulk tissue RNA-sequencing data

**Fig. 3:**
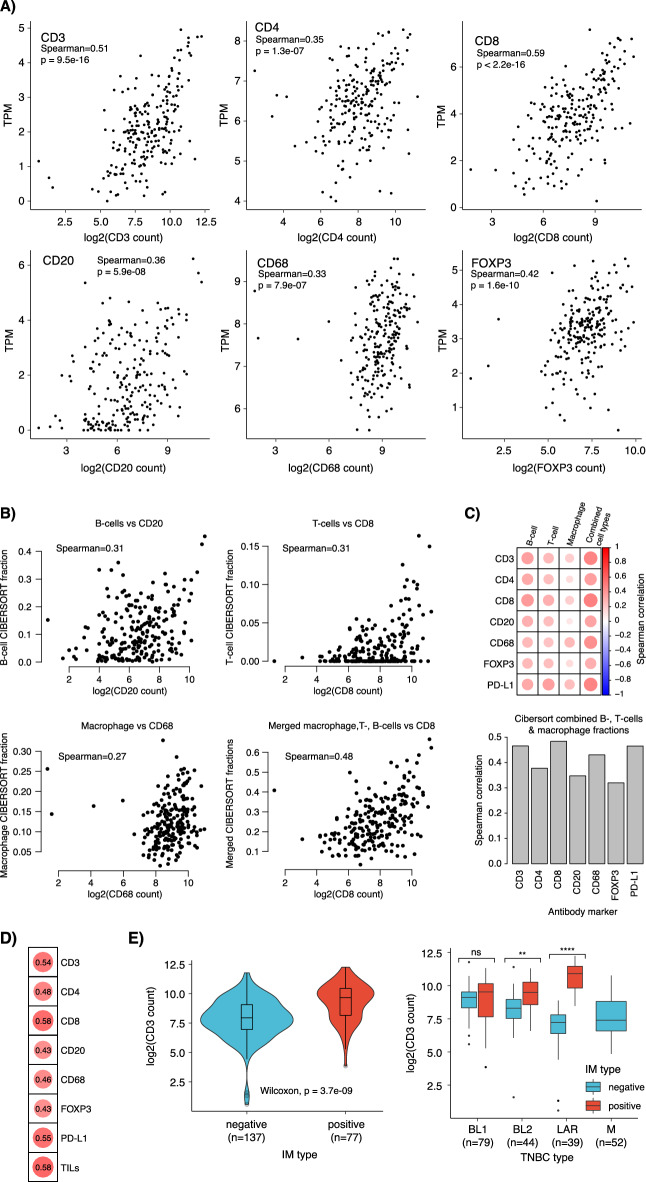
Correlation of TMArQ cell counts to RNA-sequencing data in TNBC. (**A**) Scatterplots of matched RNA-sequencing TPM-values compared to TMArQ cell counts. mRNA expression for CD3 was based on the *CD3G* gene, for CD8 on the *CD8A* gene, for CD20 on the *MS4A1* gene. (**B**) CIBERSORT immune cell proportions compared to matched cell type specific TMArQ cell counts for CD20 (B-cell marker), CD8 (T-cell marker), and CD68 (macrophage marker). In addition, a scatter plot of the summarized CIBERSORT fraction of B-cells, T-cells, and macrophages versus CD8 cell counts are shown. (**C**) Top panel shows a Spearman correlation heatmap of analysed IHC markers versus individual CIBERSORT immune types, as well as the summarized fraction of the latter. Bottom panel shows the specific Spearman correlations of the analysed IHC markers versus the summarized CIBERSORT immune faction. (**D**) Spearman correlation of rank scores for a gene expression immune metagene^21^ versus TMArQ mean cell counts (mean counts of both cores/sample). (**E**) TMArQ CD3 cell counts versus the Lehmann IM subgroup definition (left) and the Lehmann mRNA subtypes (TNBCtype-4^12^) stratified by IM status (right). Pair-wise *p*-values were computed using Wilcoxon’s test. Log transformation of TMArQ counts was performed before analyses.

Gene expression profiling has been used extensively to characterise TNBC and derive proposed molecular subtypes (see for^11^ review) but also for TIME profiling. Thus, it appears relevant to assess how actual in situ cell counts compare to bulk mRNA profiling data of matched tumours. To investigate how TMArQ cell counts matched gene expression data, we therefore compared our pipeline’s output to (1) matched bulk tissue RNA-sequencing data for analysed marker genes (TPM-values), (2) in silico estimated cell-type proportions by CIBERSORT from RNA-sequencing data (representing RNA-based estimates of TIME composition), and (3) proposed gene expression subtypes in TNBC, including an immunomodulatory (IM) class derived from mRNA expression. Figure 3A shows the automated cell counts from the TMA core versus corresponding RNA-sequencing TPM values for the analysed immune markers. The strongest correlations were observed for the CD8 and CD3 markers with Spearman correlations of 0.59 and 0.51, respectively. This indicates that cell counts, and bulk gene expression values are in agreement with each other. Regarding potential sources of confounding, we note that the RNA-sequencing and TMA data was generated from different pieces of the same tumour sample and are therefore sampled several millimetres apart.

Next, we compared the automated cell counts to the abundance of in silico estimated cell types (B-cells, T-cells, macrophages) by the CIBERSORT deconvolution method applied to the bulk RNA-sequencing data. Notably, Fig. 3B shows a positive Spearman correlation of only approximately 0.3 between the in silico CIBERSORT cell type fractions computed from bulk tumour tissue and matched TMArQ in situ cell counts, a finding that extended to all analysed IHC markers as shown in the correlation heatmap in Fig. 3C. Similarly, we also observed low correlations for all markers when using the ratio of stained cells/total number of cells per core versus CIBERSORT estimates (Supplementary Fig. 3). Notably, when merging the CIBERSORT cell type fractions into a combined sum, the correlation increased substantially compared to all in situ cell counts as exemplified for CD8 in Fig. 3B and for all markers in Fig. 3C. To further investigate this finding, we compared CIBERSORT B-cell, T-cell, and macrophage estimates versus the same RNA-sequencing data as for the cell type marker genes shown in Fig. 3A. Here, we observe that Spearman correlations for the RNA-sequencing TPM estimates versus individual CIBERSORT estimates were at best in the same range as for antibody CD8 and CD3 cell counts versus CIBERSORT estimates (i.e., around 0.6 in Spearman correlation, Supplementary Fig. 3). Moreover, correlation estimates for individual genes (TPM) increased notably when compared to a summarized CIBERSORT cell type fraction (up to Spearman correlation 0.8, Supplementary Fig. 3), which is in line with the findings for the cell counts (shown in Fig. 3C). To further compare our automated immune marker cell counts we also correlated them to a rank-based general immune metagene score based on mRNA expression levels of positively correlated immune-associated genes^21^. This comparison demonstrated a high correlation between TMArQ counts and general gene expression-based rank-scores for particularly CD3 and CD8, equivalent to TILs (Fig. 3D).

To assess immune marker expression in proposed molecular subtypes of TNBC, we compared TMArQ CD3 (a pan lymphocyte marker) cell counts with the Lehmann immunomodulatory (IM) classification and tumour classification by the four updated Lehmann TNBC subtypes (TNBCtype-4: BL1, BL2, M, and LAR)^12^. The IM-positive class showed overall the highest abundance of CD3 + cells when analysed as a separate entity (Wilcoxon’s test p = 3.7e−9) and within the four Lehmann subtypes when used as a sub stratification (Fig. 3E). For the latter, we make several interesting observations. Firstly, the BL1 subtype appears to have similar CD3 + counts irrespective of IM-class, while the BL2 class is a mix of potentially immune warm and cold tumours. In contrast, the M (Mesenchymal) subtype appears to have an immune cold phenotype, with no IM-positive cases and correspondingly low automated CD3 + counts. For the LAR subtype, which has been proposed as a more immune cold phenotype compared to BL1 and BL2^22,23^ IM-positive cases were associated with significantly higher automated CD3 + counts than IM-negative samples. These findings suggest that lymphocyte presence as measured by our pipeline and immune classification of TNBC by mRNA expression subtypes agree with each other. Importantly though, our in situ analysis suggests clear differences in immune cell abundance depending on the underlying mRNA-based TNBCtype subtypes.

### Immune marker expression variability within and between TNBC tumours

To analyse intratumour variability in immune marker expression in TNBC we assessed TMArQ output for core-to-core variability for markers, marker-to-marker variability, and combined patterns of marker expression versus clinicopathological variables and molecular classifications based on matched RNA-sequencing and WGS.

**Fig. 4:**
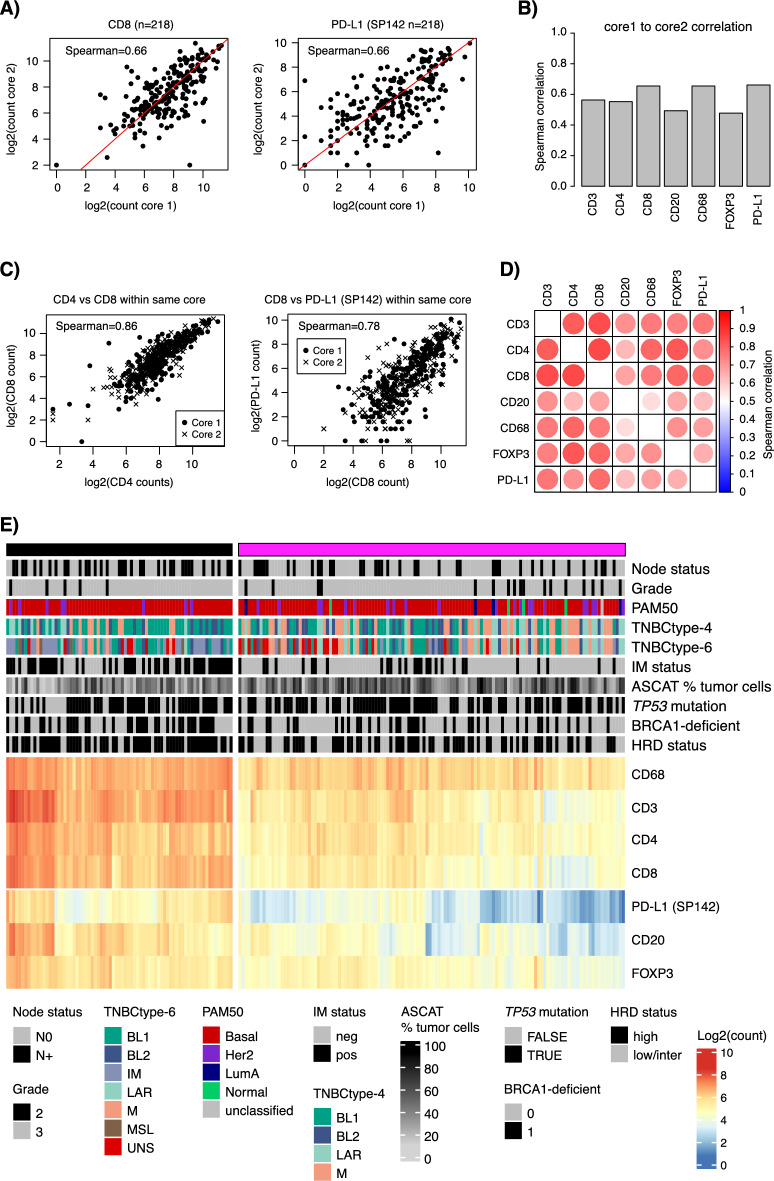
Comparison of TMA core-to-core, marker-to-marker, and marker co-expression patterns for immune markers in TNBC. (**A**) Core-to-core variability for TMArQ cell counts across 218 cases in the TMA for CD8 (left), and PD-L1 (SP142 antibody) (right). (**B**) Summarized Spearman core-to-core correlations for cell counts across all samples and markers. (**C**) Marker-to-marker correlation within cores (stacks of single-plex stains) for TMArQ cell counts from CD4 vs CD8 (left) and CD8 vs PD-L1 (SP142 antibody) (right) for all cases and cores. Spearman correlation is calculated on the merged set of data from both cores. (**D**) Spearman correlation heatmap of all marker-to-marker correlations within cores. Correlation is calculated based on the merged set of data from both cores. (**E**) Clustered co-expression matrix of average TMArQ cell counts (mean of cores/sample) for all markers and samples. Clustering was performed using Euclidian distance and complete linkage and samples were divided into two main clusters. Sample annotation track include tumour status by tumour grade, lymph node status (N0/N +), PAM50 molecular subtypes, proposed TNBC gene expression subtypes (TNBCtype-4 and TNBCtype-6), mRNA derived immunomodulatory subtype (IM) status, and WGS determined tumour cell content percentage by ASCAT, *TP53* mutation status, *BRCA1*-deficiency status (mutation or promoter hypermethylation inactivation), and HRD-status. Log transformation of TMArQ counts was performed before analyses.

The TNBC TMA used in this study contained two 1 mm cores for every sample taken from the same FFPE block, with each core targeting different tumour rich areas. Figure 4A illustrates analysis of core-to-core variability for the CD8 and PD-L1 (SP142) antibodies, while Fig. 4B summarises all markers. Overall, core-to-core Spearman correlations ranged between 0.48 and 0.66, with lower values for CD20 and FOXP3. Next, we analysed the correlation of automated cell counts between different markers within the same core (i.e., stack of single-plex stains). Here, we observed a comparably higher Spearman correlation between different antibodies within the same core. Figure 4C illustrates the correlation of CD8 to CD4 and PD-L1 cell counts within the same core, while Fig. 4D summarizes this for all marker combinations.

Next, we applied hierarchical clustering to average cell counts to explore between-sample and marker associations. Figure 4E shows the resulting two main clusters. Here we observed a general co-expression of immune markers in samples, consistent with the high within-core correlations between antibodies (Fig. 4D). Moreover, we observe a higher proportion of lymph node-positive tumours and mRNA-classified IM-positive tumours in the sample cluster with generally higher cell counts (Fig. 4E, left cluster) (Fisher’s exact test *p* = 0.02 for lymph node status, and p = 7e−8 for IM-status). For genetic tumour characteristics such as *TP53* mutation status, HRD-status, *BRCA1*-deficiency due to biallelic inactivation by mutations (somatic/germline and LOH) or promoter hypermethylation it is evident in Fig. 4E that substantial proportions of tumours are found in both immune groups. For HRD-positive cases (HRD-high), proportions between heatmap clusters in Fig. 4E were 54% and 46% respectively, despite a similar tumour mutational burden between groups (Wilcoxon’s test *p* = 0.21, Supplementary Fig. 4A). Thus, while HRD-positive TNBCs have a generally higher in situ cell count than HRD-negative tumours, considerable heterogeneity in immune cell infiltration levels exists within the HRD-positive tumour group (Supplementary Fig. 4B).

### Non-immune cell quantification and in silico merged antigen expression patterns

**Fig. 5:**
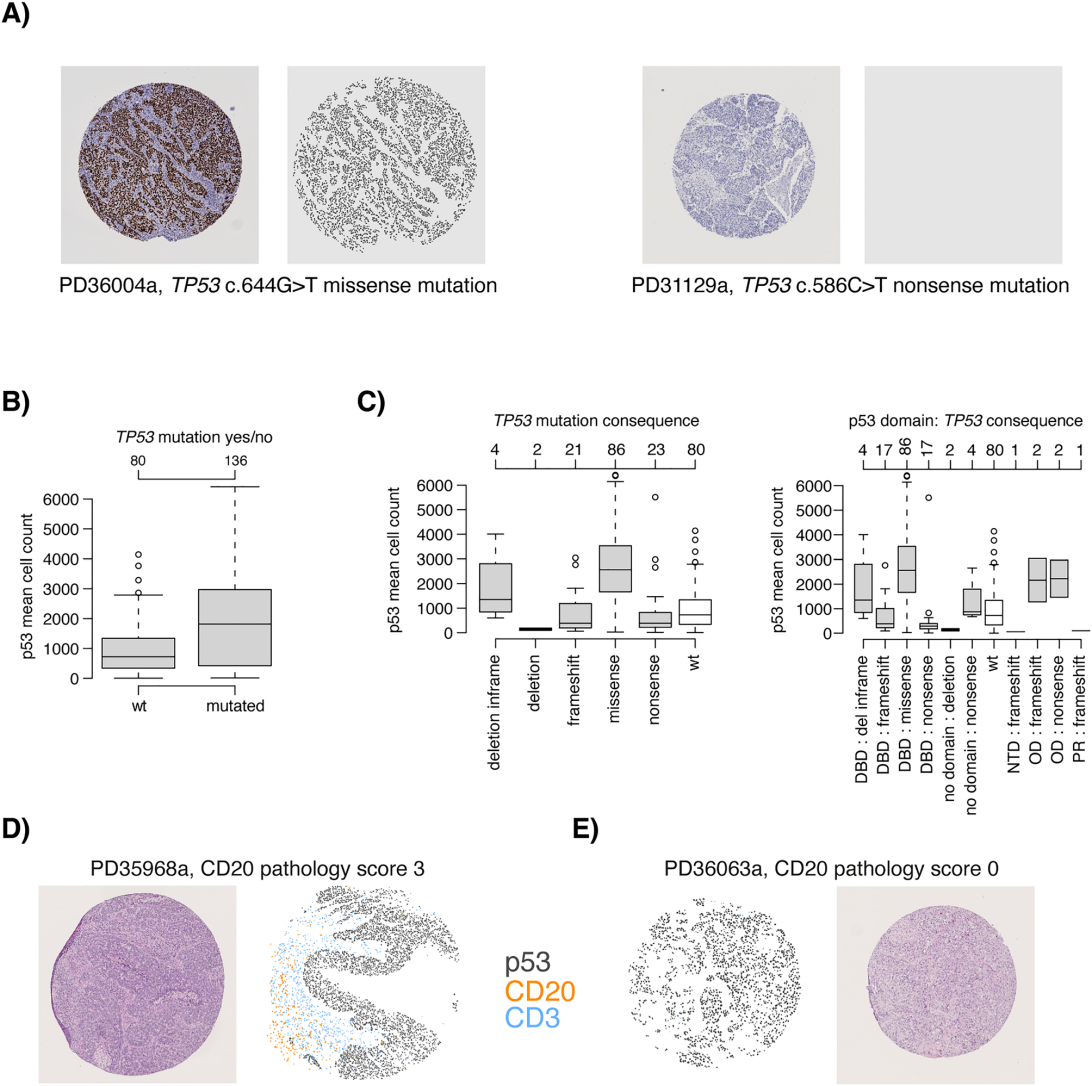
In silico merged composite images of single-plex IHC stains and application to p53 expression. (**A**) Left panel shows high p53 protein expression in a TMA core for tumour PD36004a with an c.644G > T missense *TP53* mutation. Right panel shows the low p53 protein staining in a TMA core for tumour PD31129a with a c.586C > T nonsense *TP53* mutation. (**B**) TMArQ cell counts for p53 versus WGS *TP53* mutation status (wild type/mutated). (**C**) Left panel shows cell counts for p53 versus *TP53* mutation consequence. Right panel shows the p53 cell counts for the combination of mutation consequence and p53 protein domain (obtained from^62^). In both panels, the *TP53* wildtype (wt) group is shown in white for reference. In panels B and C, the average p53 counts of both TMA cores are shown. DBD: DNA binding domain, NTD: N-terminal transactivation domain, OD: oligomerization domain, PR: proline-rich domain. (**D**) In silico merged composite stains for CD20, CD3, and p53 for tumour PD35968a. This tumour is HRD-positive by WGS and has a CD20 pathology score of 3 + (highest score). Cell objects for each marker are assigned a specific colour as indicated in the legend. **E**) In silico merged composite stains for CD3, CD20, and p53 for tumour PD36063a which is HRD-positive by WGS and has a CD20 pathology score of 0 (lowest score).

The first step of TMArQ for analysing TMA cores involves circle detection and coordinate normalization. This step enables us to place all subsequently detected cell objects on a common coordinate grid, which allowed us to test the concept of creating in silico merged multiplexed images of separate IHC stains for our single-plex markers. For studying tumour-immune interactions, it is essential to distinguish cancer cells from normal epithelial cells and immune cells. In addition to using epithelial cell markers, somatic alterations may infer protein expression that can be used to identify malignant cells. An example of the latter is mutational inactivation of the tumour suppressor gene *TP53*, a frequent event in TNBC, which can confer protein overexpression in mutated cells^24,25^. The type of mutation can conceivably influence the expression pattern. As mutational inactivation occurs only in the malignant cell, p53 protein expression could be used to identify and distinguish malignant cells across the core surface. We stained our TMAs for p53 and quantified positive cells using our automated pipeline for comparison versus *TP53* mutational status determined by WGS. Figure 5A (left panel) shows an example for a *TP53* mutated tumour with high p53 antibody staining expression displaying a clear separation between the malignant and non-malignant cells. *TP53* mutated tumours indeed had higher stained cell counts across the cohort (Fig. 5B), although protein expression in the *TP53* mutated subset was highly heterogenous compared to the *TP53* wildtype tumours. This heterogeneity may be because not all *TP53* mutations result in high p53 protein staining, as evident for another *TP53* mutated tumour shown in Fig. 5A (right panel). Stratification by *TP53* mutation consequence and affected p53 protein domain illustrated how the in situ p53 cell counts varied depending on the variants’ consequences and positions (Fig. 5C). For instance, *TP53* deletions and *TP53* nonsense and frameshift mutations result in a typically lower p53 IHC-based cell count as these variant types would typically not generate an expressed protein. Notably, p53 cell counts were especially low for tumours with nonsense mutations within the DNA binding domain (DBD) of the protein (Fig. 5C). Illustrating our findings, heterogeneous p53 staining in both *TP53* mutants, but also *TP53* wildtype cases, is a well-known phenomenon in gynaecological cancers like high grade serous carcinomas^26,27^.

Given that epithelial cells can be demarked by either pan-cytokeratin stainings, or somatic alterations like *TP53* when present, this may allow for exploration of tumour-immune interactions using image overlays. We tested this by overlaying immune cell markers with p53 stains as a proof of concept of an in silico merged multi-marker application of the pipeline. As illustrated in Fig. 5D and E, this approach enables the analysis of spatial interactions between markers of different cell types in TNBC based on TMArQ output.

### Immune cell counts and patient outcome in TNBC

**Fig. 6:**
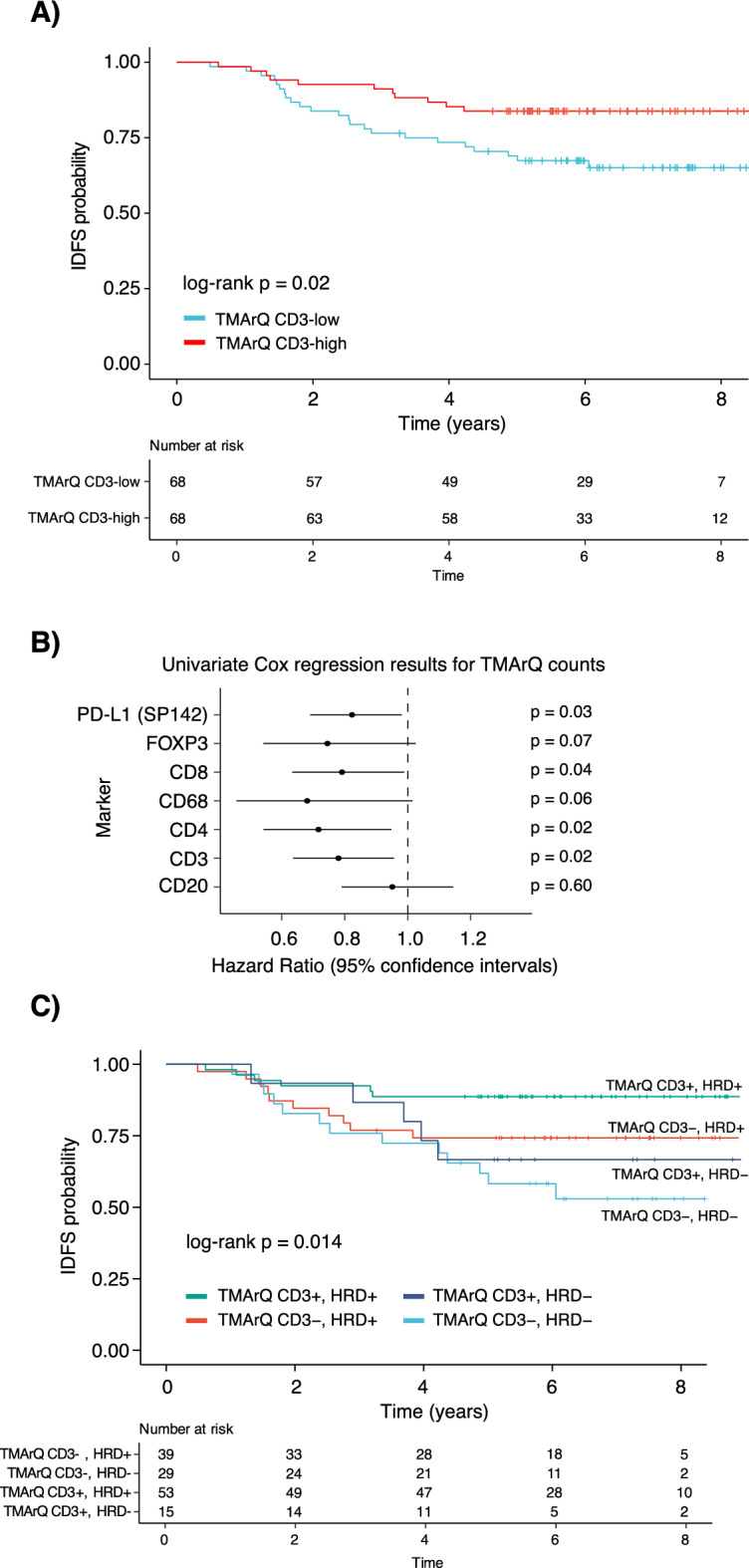
Association of TMArQ cell counts with patient outcome after adjuvant chemotherapy in TNBC. (**A**) Kaplan–Meier plot of IDFS as clinical endpoint for log2-transformed TMArQ CD3 cell counts (mean value of both cores) stratified into two groups (low/high) based on the median cell count in TNBC patients treated with adjuvant chemotherapy. The *p*-value was calculated using the log-rank test. (**B**) Forest plot illustrating hazard ratios with 95% confidence intervals from univariate Cox regression analysis of log2-transformed TMArQ cell counts as continuous values for each immune marker. Each tumour was represented by its average cell count of the two cores. (**C**) Kaplan–Meier plot of IDFS as clinical endpoint for patients treated with adjuvant chemotherapy stratified by TMArQ CD3-low/high and WGS-based HRD-status (positive/negative). The *p*-value was calculated using the log-rank test.

The TIME, as measured by for example TILs or mRNA expression of immune-related genes has repeatedly been associated with improved outcome in early-stage TNBC patients treated with and without (neo)adjuvant chemotherapy^5–7,28^. Considering the correlation of TMArQ cell counts with different pathology scores and RNA-sequencing estimates we analysed whether the automated cell counts also carried prognostic information for TNBC patients in our cohort treated with adjuvant chemotherapy. As seen in Fig. 6A for the CD3 marker, dividing the automated CD3 cell counts into two groups (low/high) based on the median observed cell count was associated with a difference in invasive disease-free survival (IDFS), with the CD3-high group showing improved patient outcome. This difference was also statistically borderline nonsignificant in multivariate analysis using patient age, tumour size, lymph node status, and tumour grade as covariates (CD3 hazard ratio (HR) 0.49, 95% confidence interval (CI) 0.24–1.013, *p* = 0.054). To expand this analysis, we performed univariate Cox regression analyses for all seven immune markers, using cell counts as continuous values in the Cox models (Fig. 6B). In agreement with the overall aspect of an immune response being associated with improved outcome in TNBC, we observed hazard ratios < 1 for the group of high versus low cell counts for all markers, with statistically significant results for the CD3, CD4, CD8, and PD-L1 markers, but not FOXP3, CD68, and CD20. In comparison, univariate Cox regression for pathologist-estimated whole slide TILs (with TILs as continuous values) showed a hazard ratio of 0.963 with a 95% CI 0.941–0.985 and a *p*-value of *p* = 0.001. Moreover, a median split of TIL scores (binary low/high groups using all cases) was significantly associated with IDFS in chemotherapy-treated patients with the high group having superior IDFS (log-rank *p* = 0.0001). Together, this demonstrates that output from the automatic pipeline is consistent with a general prognostic theme of immune response in TNBC and suggests that the signal of a clinically relevant immunological tumour response in TNBC may be captured in a small sample such as a 1 mm TMA core.

In TNBC, HRD-status has also been proposed as a prognostic factor in addition to immune response^3^. Based on the marker co-expression analysis shown in Fig. 4E, which revealed that HRD-positive tumours (by WGS) were present in both the immune-high and immune-low clusters, we constructed a 4-tier classification based on genetic HRD-status and CD3-low/high cell counts. We then evaluated the association between this stratification and IDFS in adjuvant chemotherapy-treated patients (Fig. 6C). Notably, an excellent outcome was observed for the HRD + /CD3-high combination, while HRD + /CD3-low cases had a similar (worse) prognosis as HRD-/CD3-high cases. The poorest outcome was observed for HRD-/CD3-low cases, with a long-term IDFS of approximately 50%. Together, this analysis demonstrates how quantitative measures of cell abundance can be combined with other molecular or clinicopathological variables to explore novel associations and identify subsets of patients with different outcomes following therapy.

### TMArQ immune cell counts versus genetic alterations in the context of potential drivers of immunogenicity in TNBC

Based on available WGS data for the SCAN-B TNBC cohort we analysed pathologist-derived TIL-estimates and TMArQ immune cell counts for the seven immune markers versus aggregated genetic alterations (single substitution classes, indel types, mutational load), and mutational and structural rearrangement signature exposures to explore correlations with immune TME status (Supplementary Fig. 5). Overall, only weak correlations were observed in the total cohort (Supplementary Fig. 5A–E), with the strongest correlations observed for mutational signature SBS3 (associated with BRCAness) and SBS5 (Supplementary Fig. 5D). Considering that SBS3 is associated with HRD, and that HRD-high cases displayed generally slightly higher immune scores (Supplementary Fig. 4) we performed the same analysis for mutational signatures only in HRD-high TNBCs. In this analysis, the positive correlation of SBS3 disappeared and the negative correlations of SBS5 with different immune counts were drastically reduced (Supplementary Fig. 5F).

### Application of TMArQ in other malignancies

To illustrate TMArQ’s use outside of the TNBC cohort we applied the pipeline to existing CD3 IHC data from one reported bladder cancer study (n = 289 patients)^29^ and one malignant melanoma cohort (n = 259 patients)^30^.

**Fig. 7:**
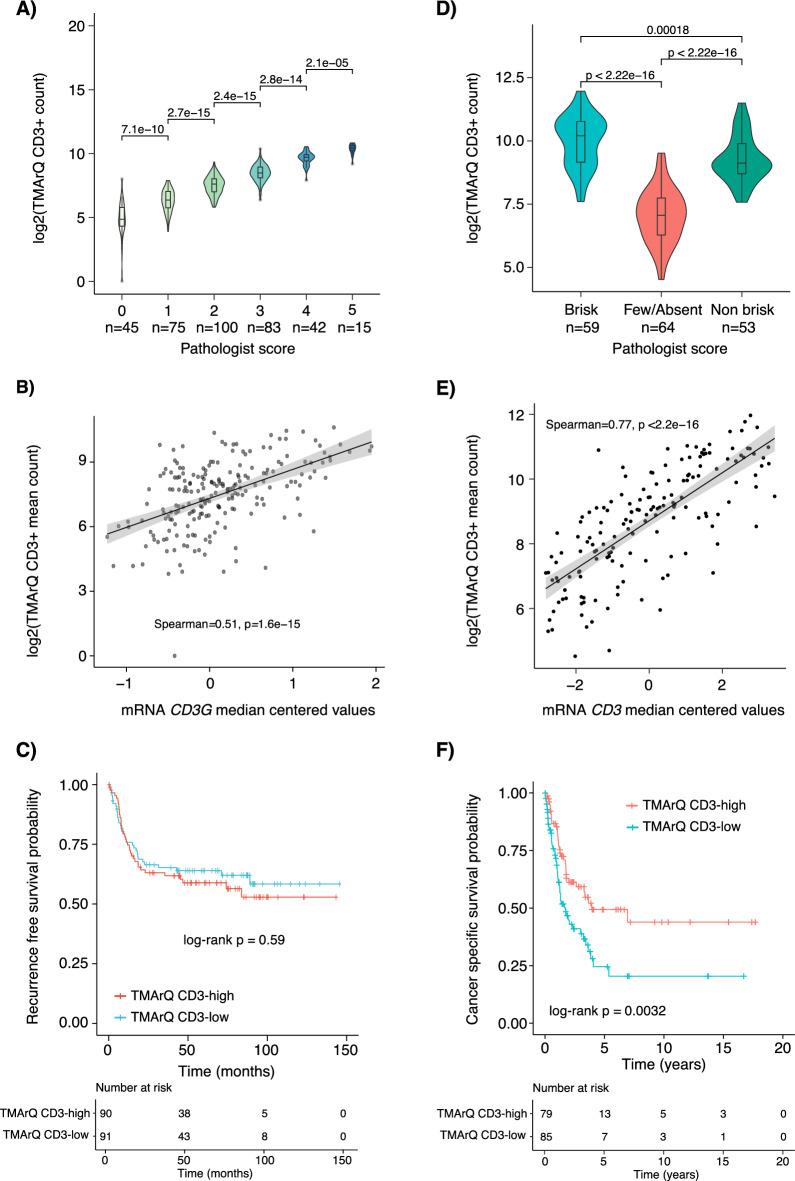
Application of TMArQ to single-plex CD3 IHC data from bladder cancer and malignant melanoma. (**A**) TMArQ CD3 counts versus human grading of CD3 expression into five bins in TMA data (n = 360 cores) from 289 bladder cancers. (**B**) TMArQ CD3 mean counts for 211 patients with matched *CD3* (*CD3G*) mRNA expression levels. (**C**) Kaplan–Meier plot of bladder cancer patients without adjuvant chemotherapy stratified by their median TMArQ CD3 counts (average core score per patient) into high and low groups using recurrence free survival as endpoint. *P*-value calculated using the log-rank test. (**D**) TMArQ CD3 mean counts versus Brisk pathology grades in 176 cores. (**E**) TMArQ CD3 mean counts versus matched *CD3* mRNA expression levels in the malignant melanoma cohort for tumours with available mRNA data. (**F**) Kaplan–Meier plot of malignant melanoma patients stratified by their median TMArQ CD3 counts into high and low groups using cancer specific survival as endpoint. The stratification was based on the median of the average CD3 count per patient (i.e. the average count across cores per patient) using the entire cohort of patients. *P*-value calculated using the log-rank test. Log transformation of TMArQ counts was performed before analyses.

For the bladder cancer cohort, we first compared TMArQ cell counts to an available pathology grading score of 1–5, with 5 indicating the highest infiltration of CD3 + cells (Fig. 7A). Similar to the CD20 comparison in TNBC, a good separation and agreement between pathology scores and automated cell counts were observed. Moreover, TMArQ cell counts were well correlated with *CD3* mRNA expression in the bladder cohort (Spearman correlation of 0.52, Fig. 7B). Next, we tested whether the TMArQ CD3 counts also carried prognostic information in the whole patient cohort. As seen in Fig. 7C, dividing the TMArQ CD3 cell counts for all cases into two groups (low/high) based on the median observed cell count was not significantly associated with better recurrence-free survival, consistent with findings in the original study in which a prognostic association was found only in a tumour subset^31^.

For the melanoma cohort we first compared TMArQ cell counts to three pathology classes: (1) brisk, (2) non-brisk, and (3) few/absent, which respectively indicate the presence of TILs across the entire tumour base, only focally, or not infiltrating to not at all present in the sample^32^. Consistent with TNBC and bladder cancer we observed a good separation of CD3 cell counts for the different brisk scores and overall higher cell counts in the brisk vs. the non-brisk samples (Fig. 7D). The few-absent score has the lowest cell counts of all categories, which is in line with expectations. Correlation of TMArQ CD3 cell counts to normalised *CD3* mRNA expression showed a high correlation in the melanoma cohort (Spearman correlation of 0.77, Fig. 7E). Finally, we performed a survival analysis based on TMArQ cell counts in the melanoma cohort and found a strong association of higher counts with better cancer-specific survival in line with the original report^30^ (Fig. 7F).

## Discussion

In the current study we developed an open-source, automated digital analysis pipeline (TMArQ) for stained cell scoring of tumour tissues using image data from single-plex IHC stains. TMArQ cell count scoring was validated versus e.g., pathological estimates, gene expression data, *TP53* mutational status, and patient outcome in three TMA tumour cohorts, but also compared to output from a commonly used digital image analysis software (QuPath). Moreover, we used TMArQ to more deeply illustrate how the immune TME in TNBC could be analysed versus different molecular classifications based on mRNA subtypes, HRD-status, mutational types and mutational signatures using a unique multi-omics TNBC cohort. Together, these analyses demonstrate how automated cell counts on individual TMA cores can facilitate a reproducible, quantitative, and accessible way to generate and test hypotheses and perform integrative multi-omics analyses on large data sets.

Most of our analyses are based on multiple immune marker stainings from a TMA constructed from a molecularly very well-characterised cohort of population-representative early-stage TNBC from South Sweden 2010–2015^3,10^. While this cohort allows for in-depth comparisons versus molecular data from both RNA-sequencing and WGS, we note that the cores in the used TMA were aimed to target tumour-rich areas in the corresponding FFPE tumour tissue blocks (similar to the bladder and melanoma cohorts). As such, the TMA cores may not necessarily capture the full immune phenotype of the TME in their respective tumour. This could in part explain some of the observed differences between pipeline and pathology estimates for TILs in TNBC. In line with this, in silico sampling of TMA equivalent cores from whole slide images for Ki67 estimation in breast cancer has showed that optimal tissue sampling for IHC biomarker evaluation is dependent on the heterogeneity of the studied tissue, which may require a substantial number of TMA cores to be sampled to achieve a low error in heterogenous tumours^33^. Moreover, for each tumour case in the TNBC TMA there were two 1 mm cores and the averages of these have been used in most analyses. As demonstrated in Fig. 4, core-to-core variability exists and may in part account for discrepancies compared with estimates obtained using other methods. Interestingly, we observed a general trend towards higher marker-to-marker correlation within a core for the analysed immune markers (Fig. 4). A general trend towards higher co-occurrence of all immune cell types within a given tumour was also observed (Fig. 4E). This is not surprising considering that many of the analysed immune cell types may act together in mounting a tumour immune response. Our combined analysis of different stains from the same core (stack) for a tumour emphasizes the value of a pre-planned staining order of the TMA to facilitate in silico merging of single-plex IHC stains, to avoid issues arising from images to be merged being several micrometres physically apart in a tissue core.

To evaluate the validity of the automated IHC-based cell counts we compared TMArQ quantifications to several pathologist-derived and human grading estimates of the same stainings in TNBC (CD20 and PD-L1 SP142) as well as TIL-estimates from whole slide H&E-stains taken before TMA construction from the same FFPE block. We also evaluated CD3 staining in one bladder cancer and one malignant melanoma cohort to assess generalizability. For TILs, we observed a clear correlation between automated cell counts for the CD3 pan lymphocyte marker and pathologist estimates of TILs. As a caveat we note that IHC staining was performed on cores from tumour-rich areas that may not capture the full range of the TIME response in certain cases. In comparison, for TIL-estimations, full sections are typically recommended over biopsies in guidelines^34^, and TIL-estimates from whole slide evaluations have been reported to be higher than in matched TMA core analysis^35^. Moreover, TIL scoring based on H&E-stained specimens does not consider the various origins and functions of immune cells^36^, and like other pathology-estimated scores human TIL-scoring is associated with inter- and intra-observer variability^35–37^. In support of the validity of our approach, a notable finding is that the correlation of our automated CD3 cell counts derived from TMA cores to TIL estimates generated on the corresponding whole-slide sections by an expert pathologist was close in strength to the correlation obtained when an AI-trained TIL predictor based on deep learning methodology was applied to the same whole section H&E-stains used by the pathologist (Spearman’s rho of 0.55 vs. best AI predictor correlation of 0.63) in the study by Bai et al.^16^. Moreover, based on data from Bai et al.^16^ we note that for the same set of chemotherapy-treated patients as in Fig. 6A, our automated cell counts outperformed the AI-trained eTILs estimates in survival association. The latter was not significantly associated with IDFS when patients were split into low/high groups based on the median eTIL (log-rank *p* = 0.24, Supplementary Fig. 4C). This may indicate that simple estimates such as automated leukocyte counts on dual 1 mm TMA cores can be used to probe relevant clinical correlations in a high-throughput fashion.

Estimation of PD-L1 status has become important in cancer therapy following the introduction of immune checkpoint inhibitors (ICIs) in patient care. A problematic issue in this context is that usage of an ICI is often associated with the requirement to use a specific PD-L1 antibody as an accompanying diagnostic assay to guide treatment^38^. The SP142 PD-L1 antibody is the accompanying diagnostic assay for the PD-L1 inhibitor atezolizumab, which was first reported effective in combination with nab-paclitaxel in patients with unresectable, locally advanced, or metastatic triple-negative breast cancer in the phase 3 IMpassion130 trial^39^. This trial led to an accelerated FDA approval for atezolizumab that however was later revoked based on negative results from the IMpassion131 trial^40^. Irrespectively, for digital pipeline assessment, the SP142 assay serves as an excellent example, with a positive staining call of PD-L1-positivity set as low as 1% staining of tumour-infiltrating immune cells (ICs), compared to the scoring of PD-L1 expressing tumour cells (TCs). Figures 2A and B demonstrate that automated cell counts correspond well with pathology-derived binary classes of PD-L1-low/PD-L1-high, but also that there is a strong correlation to actual SP142 grading in percent. Here, the full dynamic range of digital analysis pipeline counts could enhance scoring and reproducibility at low expression levels compared to the challenging assessment of whether more or less than 1% staining of IC exists by the human eye. PD-L1 IC scoring is challenging for several reasons as discussed in^38^ that together may explain discrepancies observed both for the discrete class analysis and the overall correlation analysis in this study. In the near future, it is conceivable that computer image analysis algorithms could function as an orthogonal method for PD-L1 IC scoring particularly for borderline and challenging cases^38^. Finally, we also compared automated cell counts to pathology scores for CD20 in TNBC (classified into four expression bins) and for CD3 in bladder and melanoma (Figs. 2C, 7A, and D, respectively). For CD20 we observed a good agreement between cell counts and score groups, but also a particularly sharp increase for the highest score bin. This sharp increase may indicate either a potential manual bias in the scoring of the highest CD20-expressing tumours, or a biological aspect of large numbers of infiltrating CD20 + cells (e.g., as tertiary lymphoid structures) connected to some specific tumour feature. Regarding the latter hypothesis, the highest CD20 pathology group (group 3) contained 80% HRD-positive tumours, compared to 66% for group 2, 60% for group 1, and 44% for group 0. Whether this observation represents a direct consequence of a specifically rearranged tumour genome, or merely a correlative association remains to be determined through deeper molecular analyses.

In addition to comparisons to matched pathology estimates we also compared how automated IHC-based cell counts match to different outputs from matched bulk tissue RNA-sequencing, as RNA-sequencing data is often used in cancer studies to infer TIME characteristics when matched in situ data is missing (e.g. through deconvolution methods like CIBERSORT). Despite that these comparisons are: (1) made on different tumour pieces, and (2) made on selected tumour rich areas for the IHC stainings versus RNA extracted from bulk tissue without specific regard for tumour cell content, we observed strong correlations between TMArQ cell counts and matched gene expression for particularly CD3 and CD8 (Fig. 3A, 7B, and E). Notably, for these markers, Locy et al.^41^ also reported the highest agreement between TIL pathology scores and mRNA expression estimates based on NanoString analysis. Overall, a good correlation between estimates by IHC or flow cytometry and bulk mRNA expression levels (in similar ranges that we observe) has been reported for certain cell types (including T-cells, cytotoxic cells, mast cells, and macrophages) in different malignancies^42^. Bulk mRNA expression however cannot provide insights into the spatial distribution of cell types as in situ analyses can.

Several transcriptome-based cell-type quantification methods for immuno-oncology have to date been reported, of which CIBERSORT is one of the most commonly used^43^. When CIBERSORT estimated cell type proportions were compared to in situ cell counts we found an intermediate correlation for macrophages, B-cells, and T-cells between the methods for analysed antibodies (Figs. 3B and C). The correlation increased somewhat when CIBERSORT cell type fractions were summed into a more general immune cell score. Collectively, these findings imply that CIBERSORT deconvolution predominantly identifies a general trend of immune cells rather than specific cell subsets in TNBC, except for outlier cases that score highly using any method. Notably, when comparing CIBERSORT B-cell proportions versus the CD20 pathology classifications we observed increasing fractions for CIBERSORT up until the highest score category, which had a lower average CIBERSORT B-cell fraction than the second highest group (Supplementary Fig. 3). This contrasts with the pattern seen for the cell counts (Fig. 2C), suggesting that the in situ derived counts are in better agreement with manual scoring than the deconvolution method. Moreover, it should be noted that observed correlations between automated cell counts and bulk mRNA deconvolution cell estimates are likely in line with what might be expected in heterogeneous breast cancer tissue. This assertion is consistent with observations using xCell^44^ (a deconvolution algorithm similar to CIBERSORT), which showed correlations between 0.5 and 0.7 when estimated cell proportions were compared to cell sorted blood proportions. Together, this illustrates the difficulties in using in silico deconvolution methods for precise assessments of the TIME in individual tumours.

When we compared TMArQ CD3 cell counts to a more general immune classification in TNBC, the IM-class^12^, we observed a consistent pattern overall with higher CD3 quantifications in IM + tumours. The IM classification is based on a general set of immune response associated genes considering the general pathway enrichments reported by Lehmann et al.^45^. As such, the IM-signature may be less associated with the expression of a specific cell type, and more with a general immune cell load. While patients with LAR-classified tumours have been associated with both a generally immune cold TME (together with the TNBCtype M class^11^) and a generally poorer response to conventional systemic therapy^12,46^, it remains to be determined if the observation of a subset of TNBC LAR patients with high lymphocyte infiltration (reflected by high automated CD3 cell counts, Fig. 3E) translates to a difference in patient outcome. Considering the high marker-to-marker cell count co-expression we observed for individual marker pairs, we also clustered TMArQ cell counts for all analysed immune markers (Fig. 4), a possibility associated with the quantitative data generated by tools like TMArQ. This analysis revealed that most markers appeared correlated with regard to cell count levels, defining at one end an immune cold subset of TNBC tumours and at the other end an immune warm tumour subset, as reported previously^9,11^. Consistent with the established prognostic role of immune response in TNBC and the general immune marker-to-marker co-expression in TMA cores, patient stratification based on automated cell counts subdivided patients in our cohort with adjuvant systemic therapy into groups with differing prognosis in terms of IDFS (Fig. 6). This association was also observed for pathologist-derived TIL scores, but not for AI-estimated eTILs^16^. As for PD-L1 scoring, the continuous counts produced by digital analysis tools could be useful for considering threshold selection for the definition of prognosis or risk groups in a discovery-validation cohort approach.

Another observation made in our TNBC cohort based on linking the cell count co-expression analysis with genomic data was that a highly rearranged tumour genome is not equivalent to an immune warm phenotype in situ. This is exemplified by WGS-classified HRD-positive tumours in our cohort, that bear highly rearranged genomes and a high mutational load^3^. In our analyses, these tumours with similarly complex genomes display variable automated immune cell counts across all investigated immune markers (Fig. 4). Similar findings, albeit using more limited sequencing data, have also been noted by other studies^9^. Therefore, one can infer a complex interplay between DNA repair deficiency status and the immune phenotype of the TIME that may have considerable prognostic relevance. Indeed, a simple 4-tier grouping of HRD-status and dichotomized CD3 cell counts showed that patients with HRD-positive tumours with higher CD3 counts have a better IDFS after adjuvant chemotherapy compared to the other HRD/CD3 groups (Fig. 6).

Studies have reported tumour mutational burden to be associated with response to ICI based on the hypothesis that somatic variants are able to generate tumour-specific neoantigens. While the vast majority of mutations appear to have little or no immunogenic effect^47^, it has been suggested that specific types of alterations, like frameshift insertion/deletions, may represent more immunogenic variant subsets^48^. To test this, we performed a comprehensive analysis of single base pair substitutions, indels, and mutational and rearrangement signatures versus TMArQ counts of the seven immune markers in the WGS-matched TNBC cohort (Supplementary Fig. 5). In TNBC, these analyses showed only weak correlations that do not support that total numbers, specific mutation types, or mutational signatures (representative of mutational processes) are strongly correlated with total immune cell counts. The analyses demonstrated also that when considering the HRD genetic phenotype, correlations weakened further. The latter observation emphasizes that the genetic/molecular background of samples and cohort compositions needs to be considered when evaluating associations of molecular alterations to proposed immune cell subgroups of TNBC like reported in the study by Hammerl et al.^9^. Together with the analysis of p53 staining versus the presence of pathogenic *TP53* mutations (Fig. 5), these examples illustrate how somatic tumour features from other platforms can be combined with in situ TIME estimates in breast cancer to add nuance to analyses and explore novel hypotheses.

The proposed pipeline has its limitations. We did not validate the pipeline for the use on individual patients. Curation of input images still requires a human inspection to avoid technical artefacts that may affect cores with tissue folding, considerable gaps, poor staining quality or other artefacts. However, TMArQ can be easily adapted to flag potential outliers and spot cores that appear different in quality. Apart from that, the current definition of a stained cell is based on CD3 staining and may be tailored to specific markers by identifying marker specific threshold values and potentially also improved by use of Gaussian mixture models as proposed by^49^. Regarding in silico merging of different stains we note that combining stains from a stack of sections (the z-axis of a core) may result in positional shifts when transitioning across different cell layers. To some extent, careful planning of the IHC staining order in single-plex studies may mitigate this issue. Future pipeline development could include the differentiation between detection of fully positive and partially stained cells, and alternative segmentation methods to *starDist* for general cell segmentation. While currently applied exclusively to TMA cores TMArQ could also be expanded for use on whole-slide images, including scoring of virtual TMA cores from whole-slide sections. Additionally, applying this framework to multiplex stainings on e.g., the PhenoImager-platform could allow for an even greater potential for analysing cell–cell interactions.

In summary, application of an open-source, automated pipeline for IHC-based cell detection and quantification, to a molecularly well profiled primary TNBC cohort provided multiple examples of how digital analysis of multiple single-plex IHC stains can be used to target the TIME in TNBC alone but also combined with genomic data. Examples includes the known association of a general immune response with favourable prognosis in TNBC, but also an exemplification of how merging an in situ TME readout such as CD3 + cell infiltration can be combined with a genetic classification of HRD to, in an exploratory context, further identify a good-prognosis subgroup of patients. While TMArQ currently provides cell counts, the method also allows for the spatial positioning and reporting of identified cell objects, which can be used to align and merge single-plex staining experiments into composite images for potential cell–cell interaction analysis by e.g. recently reported software tools^50^. Overall, digital image analysis tools will likely facilitate the next step in TME characterization of breast cancer and other malignancies. Using a more quantitative approach to TME profiling allows for more precise and detailed integrations with omics data such as DNA methylation, gene expression profiling, and WGS, in addition to allowing unsupervised and supervised methods typically used in omics studies to be employed in multi-layered IHC data as well. In the end, such improvements to conventional IHC data can further facilitate the next step in phenotypic and integrative cancer studies.

## Methods

### TNBC patient cohort

All included patients with primary TNBC were enrolled in the Sweden Cancerome Analysis Network—Breast (SCAN-B) study (ClinicalTrials.gov ID NCT02306096)^51,52^. Ethical approval was given for the SCAN-B study (Registration numbers 2009/658, 2010/383, 2012/58, 2016/742, 2018/267 and 2019/01,252) by the Regional Ethical Review Board in Lund, Sweden, governed by the Swedish Ethical Review Authority, Box 2110, 750 02 Uppsala, Sweden as previously described^3^. All patients provided written informed consent prior to enrolment. All methods were carried out in accordance with relevant guidelines and regulations. Clinicopathological data were obtained from Staaf et al.^3^, with updated patient outcome data for IDFS of 6.51 years for censored patients. Based on the original TNBC cohort reported by Staaf et al.^3^ we identified 218 patients with available TMA data. Patients without available TMA data were excluded. Patient specific details are provided in Supplementary Table 1.

### Bladder cancer and malignant melanoma validation cohorts

Scanned single-plex CD3 IHC stains were obtained from a previously reported bladder cancer TMA study (n = 289 tumours)^29^ and one malignant melanoma TMA study (n = 259 tumours)^30^ along with matched *CD3* mRNA expression values, and patient outcome. Full details on TMA construction, IHC experiments including imaging, gene expression analysis, patient inclusion and exclusion criteria, ethical consents, and patient characteristics are available online from the original studies^29,30^. For both the bladder cancer and malignant melanoma cohorts the primary inclusion criteria for this study was available TMA IHC data with matched clinicopathological and molecular data. Patient specific details for both cohorts are provided in Supplementary Table 1.

### TNBC TMA construction, IHC staining and human evaluator grading

For 218 patients from the cohort reported by Staaf et al.^3^ TMAs (five blocks) were constructed with two 1 mm cores/tumour as previously described^10^. The TMAs were stained for p53 and a set of immune markers: CD3, CD4, CD8, CD20, CD68, FOXP3, and PD-L1 (SP142 antibody, Roche). TIL scoring on whole section H&E-slides, CD20 IHC staining on TMA slides, and PD-L1 IHC staining on TMA slides and associated pathology scorings have been reported previously^10^. Pathology scorings for CD20 and PD-L1 were performed on the same TMA stainings used for the automated pipeline, while the TIL scoring was performed on a whole slide section taken from the same FFPE tissue block prior to TMA core sampling. For PD-L1, 1% staining in tumour-infiltrating immune cells (termed IC) was used as cut-off for PD-L1 positivity in line with guidelines for the SP142 antibody. Remaining IHC stainings were performed as outlined in Supplementary Table 2. Slides were imaged using a Hamamatsu NanoZoomer S210 scanner with 20X resolution.

### TNBC RNA-sequencing and whole genome sequencing data

RNA-sequencing data, including reported PAM50 molecular subtypes by nearest centroid correlation, for included cases was obtained from Staaf et al.^53^. Expression values were summarized to transcripts per million (TPM) estimates for this study. Entrez ID from the Gencode27 metadata was used as gene identifiers. CIBERSORT^54^ immune cell deconvolution scores and Lehmann TNBCtype-4 mRNA subtypes (BL1, BL2, M, and LAR)^12^ were obtained from the study by Aine et al.^10^ for matching samples. The Lehmann IM class (negative/positive) was defined using a cut-off in correlation of 0.17 to the original IM-centroid correlation. Computed gene expression-based rank scores for an immune metagene originally defined by Fredlund et al.^21^ were obtained for matching TNBC samples included in the study by Nacer et al.^55^. In Nacer et al. rank scores were computed individually for each tumour without any normalization or data centering (i.e., single sample scores). Matched whole genome sequencing (WGS) data for analysed cases were obtained from Staaf et al.^3^, and included besides somatic alterations also mutational signature exposures (by the SigFit algorithm reported in^10^), structural rearrangement signature exposures, and HRD predictions by the HRDetect algorithm^4^.

### Quantification of immune antibodies in IHC

Single-plex IHC stains for p53 and a set of seven immune cell antibodies were analysed using the TMArQ pipeline (Fig. 1) for the TNBC cohort. In the bladder and melanoma cohorts only the CD3 marker was analysed. TMArQ allows users to specify which images should be analysed via a configuration file. Input data currently consists of png images in RGB format of individual cores extracted using standard dearraying methods. TMArQ detects the core area using Hough circle detection as implemented in *scikit-image* version 0.19.2^56^ (http://scikit-image.org)(which could be computationally intense). The detected diameter and centre of the core are recorded in a text file, which is used to centre the core in the image for downstream analysis. If no circle is detected the image is considered empty or the tissue of too poor quality (containing e.g. heavy folding or large gaps, see Supplementary Fig. 1 for examples) and is flagged, allowing it to be excluded from further analysis.

Next, hematoxylin and DAB stains are separated using colour deconvolution, as described in^57^ and implemented in *scikit-image*^56^ (http://scikit-image.org). For cell detection the pipeline uses *starDist* segmentation version 0.8.1 (https://pypi.org/project/stardist/) for general cell detection in the hematoxylin layer^58,59^. *starDist* has shown value as a cell segmentation method in independent studies^60^ and is freely available as both Python and ImageJ versions (https://pypi.org/project/stardist/ and https://imagej.net/plugins/stardist). TMArQ then determines the presence of DAB stains by thresholding the DAB layer using triangle thresholding on an image-by-image basis to reduce the effects of non-uniform staining across cores and TMAs. It then combines the coordinates of the cells detected by *starDist* and the thresholded DAB-layer for positive/negative cell detection. TMArQ places every cell at the centre of a 16 × 16 pixel grid. If the grid in the thresholded layer contains more than 10% positive pixels we consider the cell to be positive for the specific marker. This cut-off is based on the CD3 marker (Supplementary Fig. 1), showing that cut-off variations between 5 and 25% conferred little correlation differences compared to CD3 TPM expression values from RNA-sequencing or pathology estimated TIL percentages. For further detailed information on the pipeline, please refer to the project’s website listed in the Code Availability statement. The automated TMArQ IHC-based cell counts used in this study for included cohorts are available in Supplementary Table 1.

### QuPath analysis of CD3 IHC images

For comparative purposes, CD3 IHC data from the TNBC cohort was analysed using QuPath^20^ (https://qupath.github.io). To extract semi-automated cell counts on a TMA core level, a groovy script was created that captured the manually selected steps of colour deconvolution, region (core) classification, and positive cell count detection. Obtained QuPath cell counts were compared to TMArQ extracted cell counts using Spearman correlation.

### Integration of IHC quantifications with additional data

All statistical analyses were carried out in R, version 4.4 (www.r-project.org). Automated IHC-based cell counts were log2 transformed before analyses. Correlation analyses were performed using the Spearman correlation as a measure of agreement. Survival analyses were performed using the *survival* (version 3.5.8, www.r-project.org) and *survminer* (version 0.4.9, www.r-project.org) R packages with IDFS defined according to the STEEP criteria^61^ as the clinical endpoint for TNBC patients treated with adjuvant chemotherapy. For the bladder tumour cohort recurrence free survival (RFS) was used as clinical endpoint and for the melanoma cohort cancer specific survival (CSS), based on data from the original publications. Survival curves were estimated using the Kaplan–Meier method and compared using the log-rank test. Hazard ratios were calculated through univariable or multivariate Cox regression using the *coxph* R function in R version 4.4 (www.r-project.org). In multivariate analysis for the TNBC cohort standard prognostic variables in breast cancer such as patient age (years), tumour size (mm), lymph node status (N0/N +), and tumour grade (NHG) were included as covariates. Full details concerning relevant patient inclusion and exclusion criteria, endpoint definitions, and CONSORT diagram relevant for the survival analysis of the SCAN-B TNBC cohort, the bladder cohort, and the melanoma cohort are available from original publications^3,29,30^.

## Supplementary Information

Supplementary Information 1.

Supplementary Table 1.

## Data Availability

Genomic data for the TNBC cohort supporting the conclusions of this article are available in an open repository as described in the original study (see https://data.mendeley.com/datasets/2mn4ctdpxp/3). Data for the bladder cancer and malignant melanoma cohorts can be retrieved through the respective online journal versions^29,30^. The TMArQ IHC-based cell counts supporting the conclusions of this article are included within the article as Supplementary Table 1.

## Code availability

All analyses were performed using open-source software such as the R statistical language and Python. The TMArQ pipeline is publicly available at https://github.com/StaafLab/TMArQ.
