# Supplementary material for: Tumour immune characterisation of primary triple-negative breast cancer using automated image quantification of immunohistochemistry-stained immune cells
Source: Sci Rep. 2024 Sep 13;14:21417. doi: 10.1038/s41598-024-72306-1 (PMC11399404; doi:10.1038/s41598-024-72306-1)
Supplement: Supplementary file 1 — Supplementary Information 1. [file 41598_2024_72306_MOESM1_ESM.pdf]

Tumor immune characterisation of primary triple-negative breast cancer using automated image quantification of immunohistochemistry-stained immune cells

Suze Roostee[1,2], Daniel Ehinger[1,3], Mats Jönsson[1], Bengt Phung[1], Göran Jönsson[1], Gottfrid Sjödahl[3], Johan Staaf[1,2,*,**], Mattias Aine[1,*,**]

Author's affiliations
1 Division of Oncology, Department of Clinical Sciences Lund, Lund University, Sweden
2 Division of Translational Cancer Research, Department of Laboratory Medicine, Lund University, Sweden
3 Department of Genetics, Pathology, and Molecular Diagnostics, Skåne University Hospital, Lund, Sweden
4 Department of Translational Medicine, Lund University, Malmö, Sweden

Supplementary Table 2. IHC stainings performed for this study.

Antibody	Supplier	Cat no	Clone	Dilution	Pretreatment	Visualisation	Incubation time
p53	Santa Cruz	sc-126	DO-1	1:500	Dako PT-Link Buffer pH 9	Dako EnVision™Flex K8010	30 min
FOXP3	Abcam	ab20034	236A/E8	1:200	Dako PT-Link Buffer pH 9	Dako EnVision™Flex K8010	30 min
CD68	Dako/Agilent	M0814	KP1	1:1000	Dako PT-Link Buffer pH 9	Dako EnVision™Flex K8010	30 min
CD4	Dako/Agilent	M7310	4B12	1:200	Dako PT-Link Buffer pH 9	Dako EnVision™Flex K8010	30 min
CD8	Dako/Agilent	M7103	C8/144B	1:100	Dako PT-Link Buffer pH 9	Dako EnVision™Flex K8010	30 min
CD3	Dako/Agilent	M7254	F7.2.38	1:200	Dako PT-Link Buffer pH 9	Dako EnVision™Flex K8010	30 min

A)

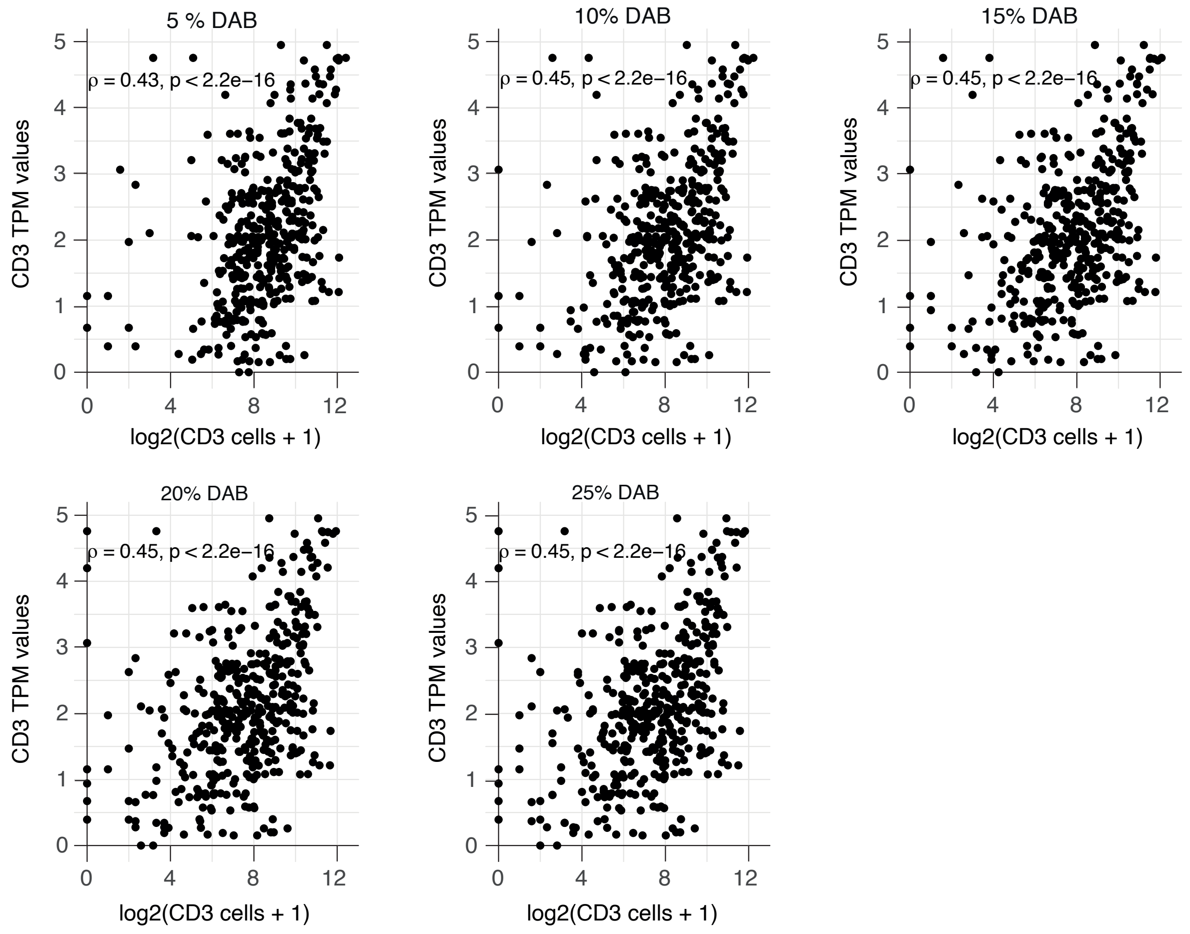

B)

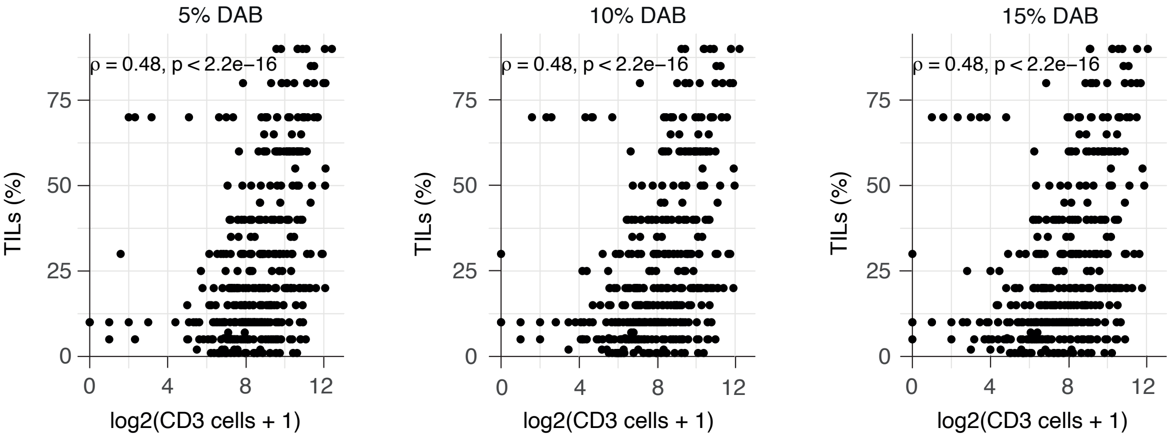

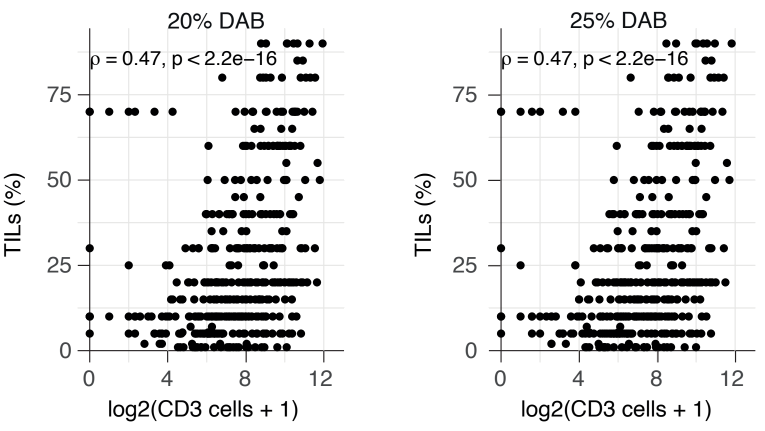

Supplementary Figure 1. CD3 positive cell count correlations under varying DAB threshold levels, and circle and segmentation examples. A) Scatter plots with Pearson correlation showing the correlations between CD3 TPM values and automated CD3 cell counts on core level. An offset of 1 was used to avoid negative log2 values for counts equal to 0. **B)** Scattter plots with Pearson correlation showing the effect of DAB threshold level between pathology estimated TIL scores and automated CD3 cell counts on core level. **C)** Three examples of Hough circle detection by TMArQ in the TNBC TMA cohort. The left example is included in data analyses and called correctly. Centre is suboptimally detected by Hough circle detection (not included in analyses) and right example should have been failed (not included in analyses). **D)** Example of starDist segmentation on different parts of the haematoxylin separated stain of a core. Coloured overlays show the starDist segmented nuclei.

C)

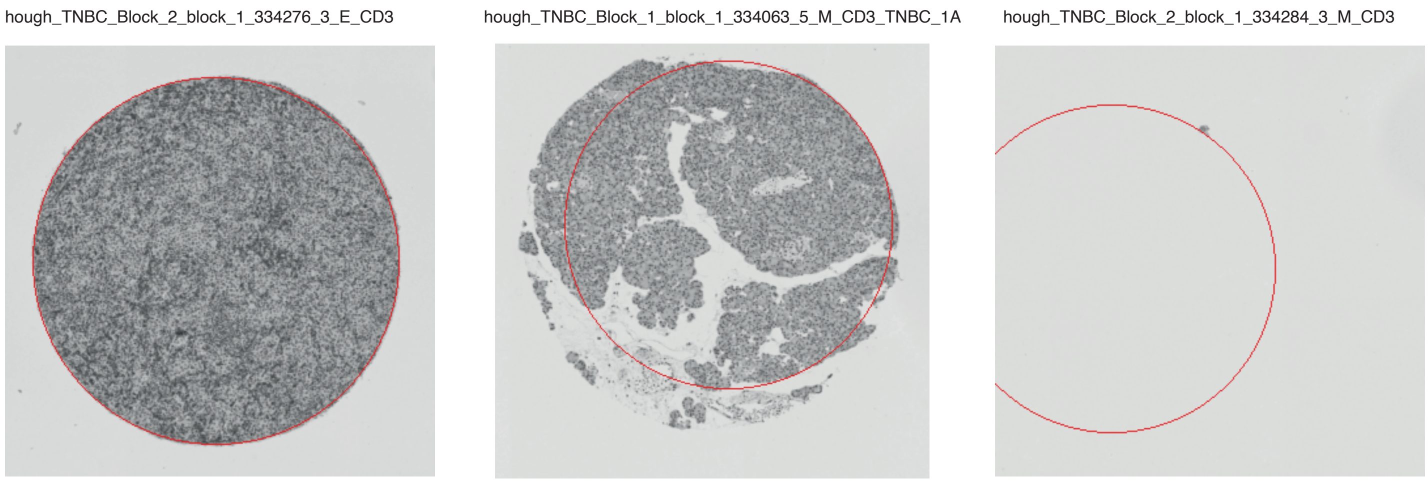
hough_TNBC_Block_2_block_1_334276_3_E_CD3
hough_TNBC_Block_1_block_1_334063_5_M_CD3_TNBC_1A
hough_TNBC_Block_2_block_1_334284_3_M_CD3

D)

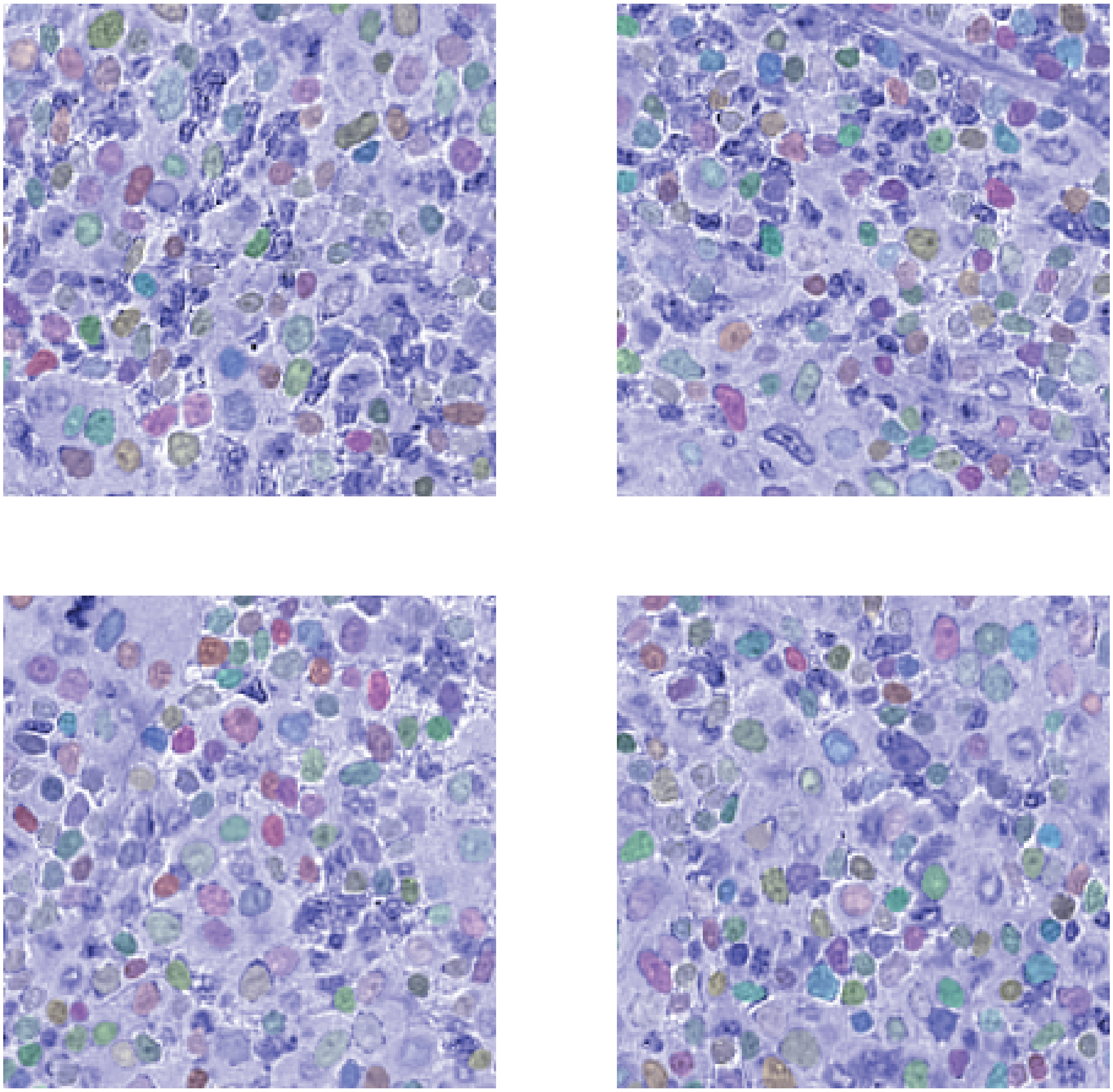

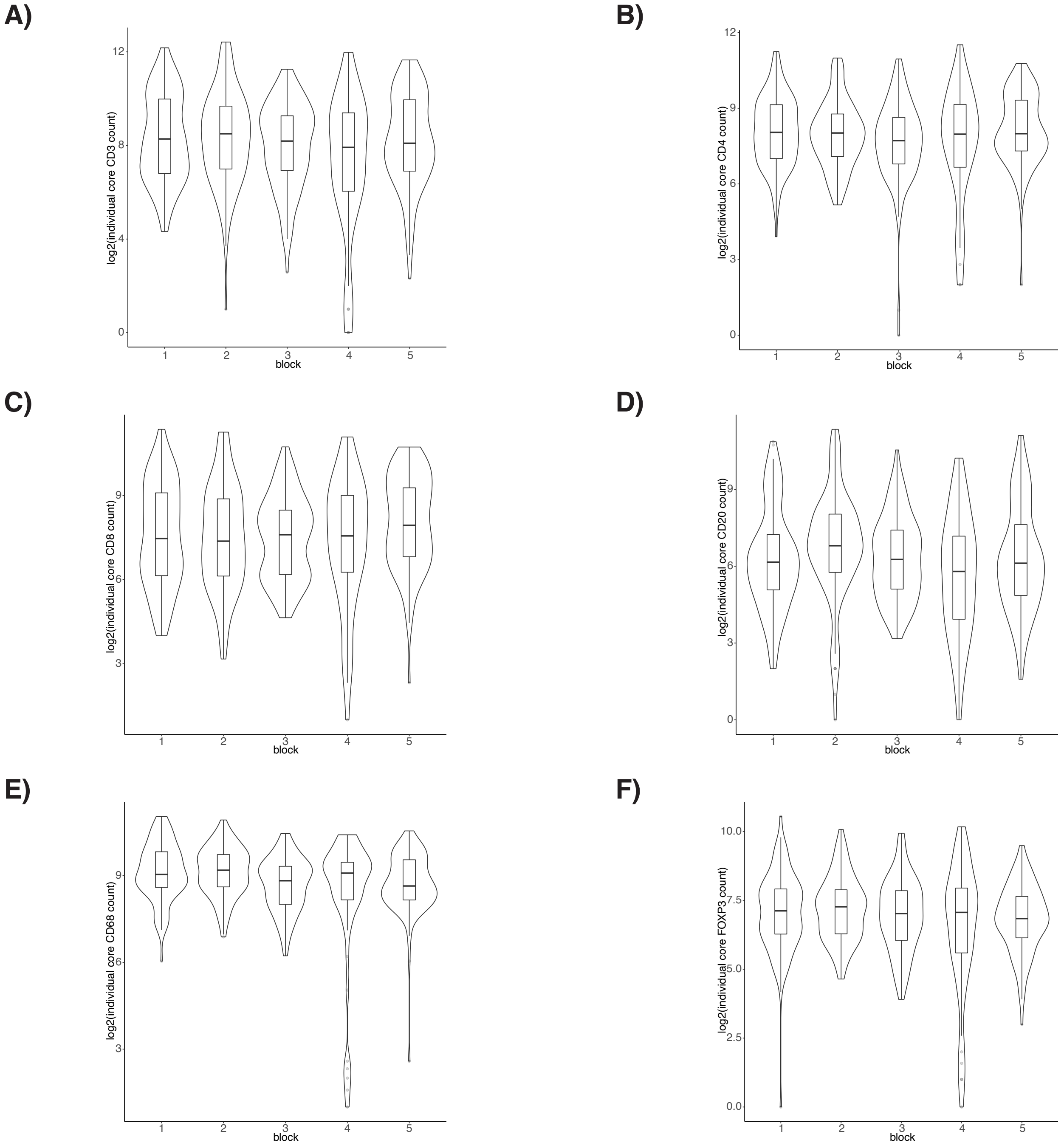

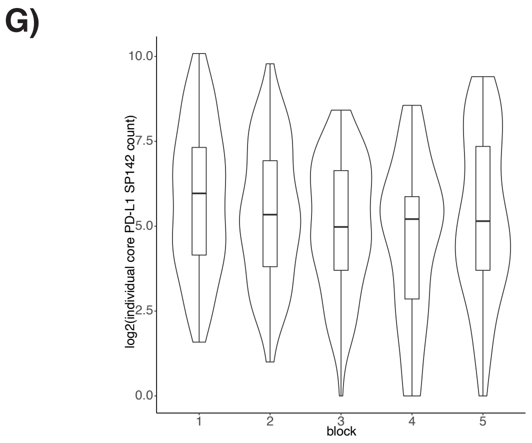

Supplementary Figure 2. Antibody cell counts per core versus TMA block for analysed immune markers. Panels A-G show violin plots for log2 transformed cell counts per TMA core versus respective block. **A)** CD3. **B)** CD4. **C)** CD8. **D)** CD20. **E)** CD68. **F)** FOXP3. **G)** PD-L1 (SP142 antibody).

A)

ratio stained / total nbr cells per TMA core

	CIBERSORT B-cell	CIBERSORT T-cell	CIBERSORT macrophage	Summed total fraction
CD3-ratio	**0.35**	**0.26**	**0.13**	**0.41**
CD4-ratio	**0.27**	**0.19**	**0.04**	**0.27**
CD8-ratio	**0.34**	**0.27**	**0.10**	**0.39**
CD20-ratio	**0.25**	**0.21**	**0.03**	**0.24**
CD68-ratio	**0.21**	**0.18**	**0.11**	**0.28**
FOXP3-ratio	**0.20**	**0.13**	**0.02**	**0.18**
PD-L1-ratio (SP142)	**0.30**	**0.29**	**0.15**	**0.38**

B)

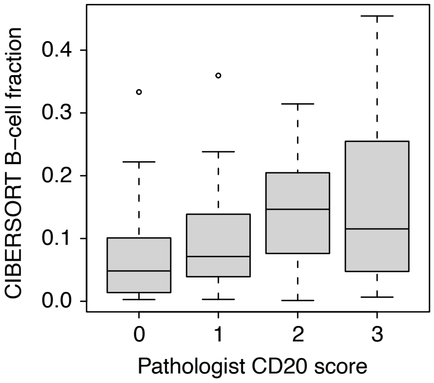

C)

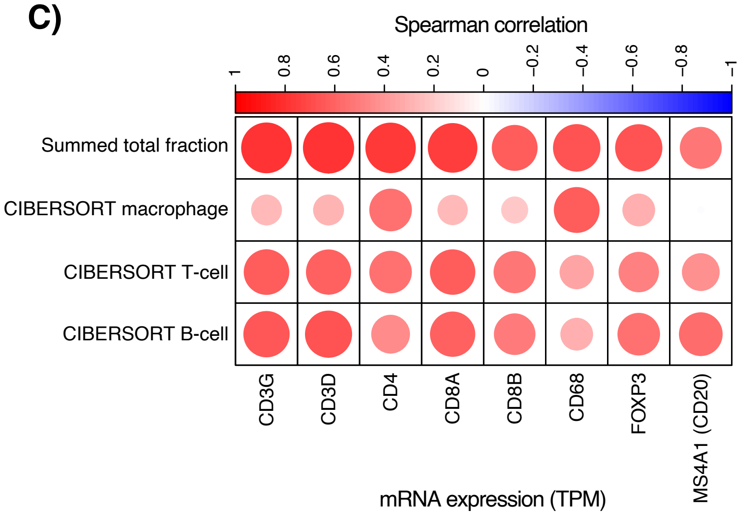

D)

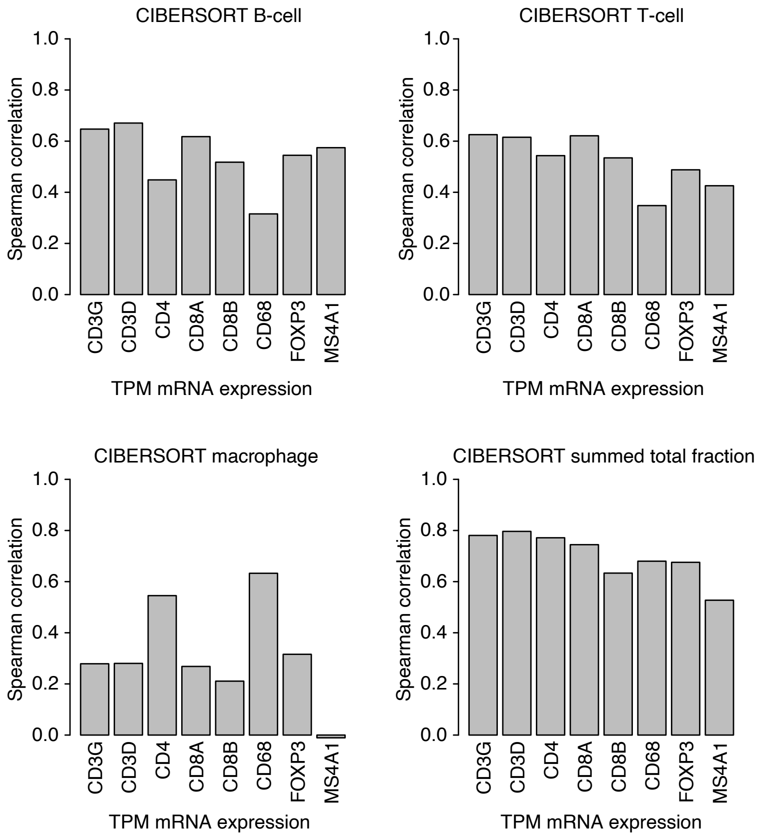

Supplementary Figure 3. Comparisons of CIBERSORT estimates versus pathologist scores and RNA-sequencing data. A) Spearman correlation table showing the correlation between CIBERSORT cell estimates and cell count ratios on a core level for investigated antibodies. Cell count ratios were computed as number of stained cells / total number of cell per core.
B) CIBERSORT B-cell estimates versus pathologist CD20 scoring. **C)** Spearman correlation heatmap showing the correlation between CIBERSORT cell estimates and TPM expression for immune genes associated with cell types. **D)** Spearman correlation bar plots for data shown in panel C.

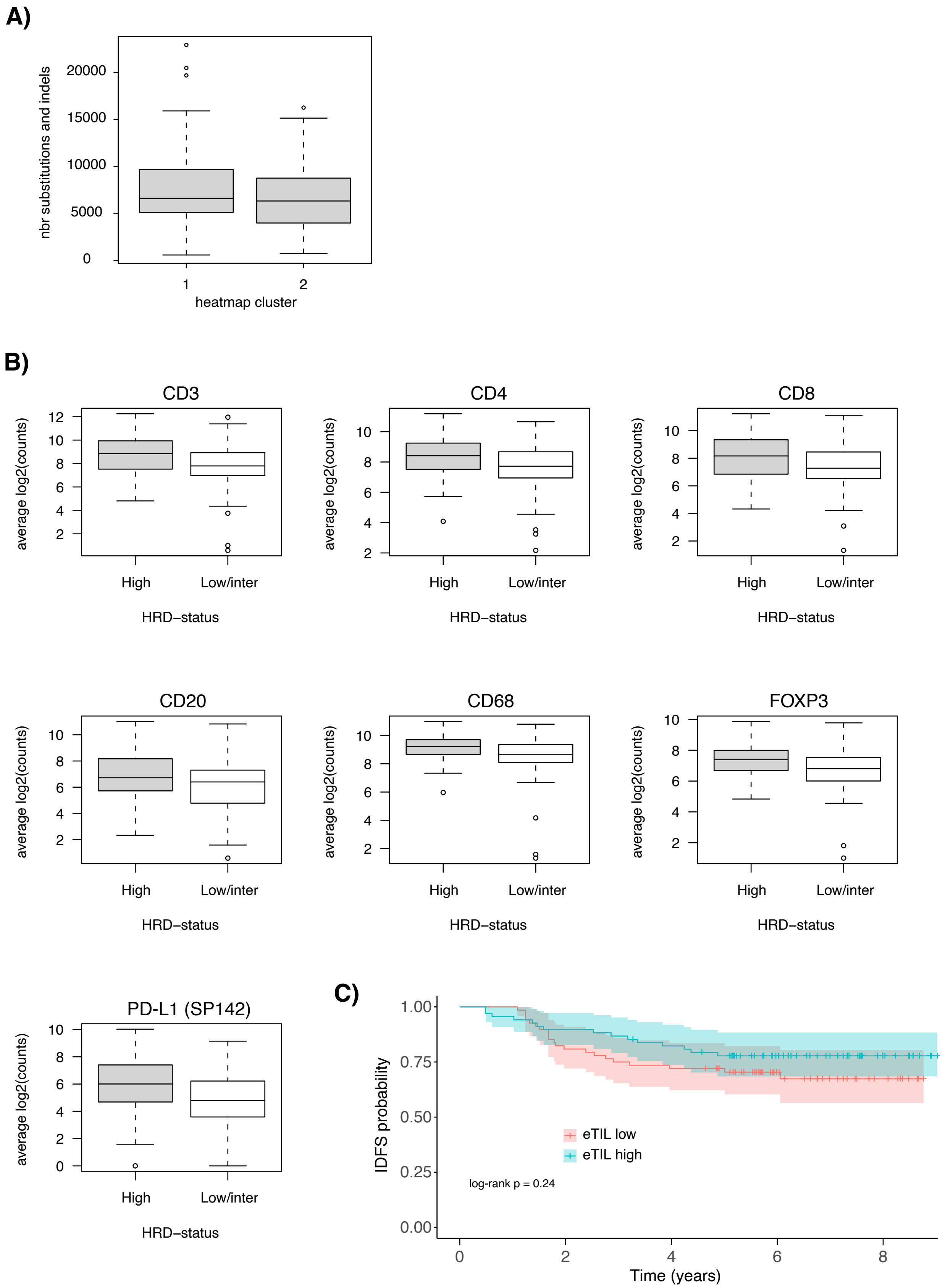

Supplementary Figure 4. Mutational burden, automated antibody cell counts per sample versus WGS determined HRD-status, and AI-TILs analyses. A) Total number of single nucleotide substitutions and indels for HRD-positive cases (by WGS) divided by the two heatmap clusters defined in main Figure 4. **B)** Automated antibody cell counts for immune markers versus HRD-status from WGS. Displayed counts represents the average value from both TMA cores for a tumor. **C)** Association with IDFS for chemotherapy treated patients for eTILS groups (low/high, by median) using data from Bai et al. on the the TMA cohort patients.

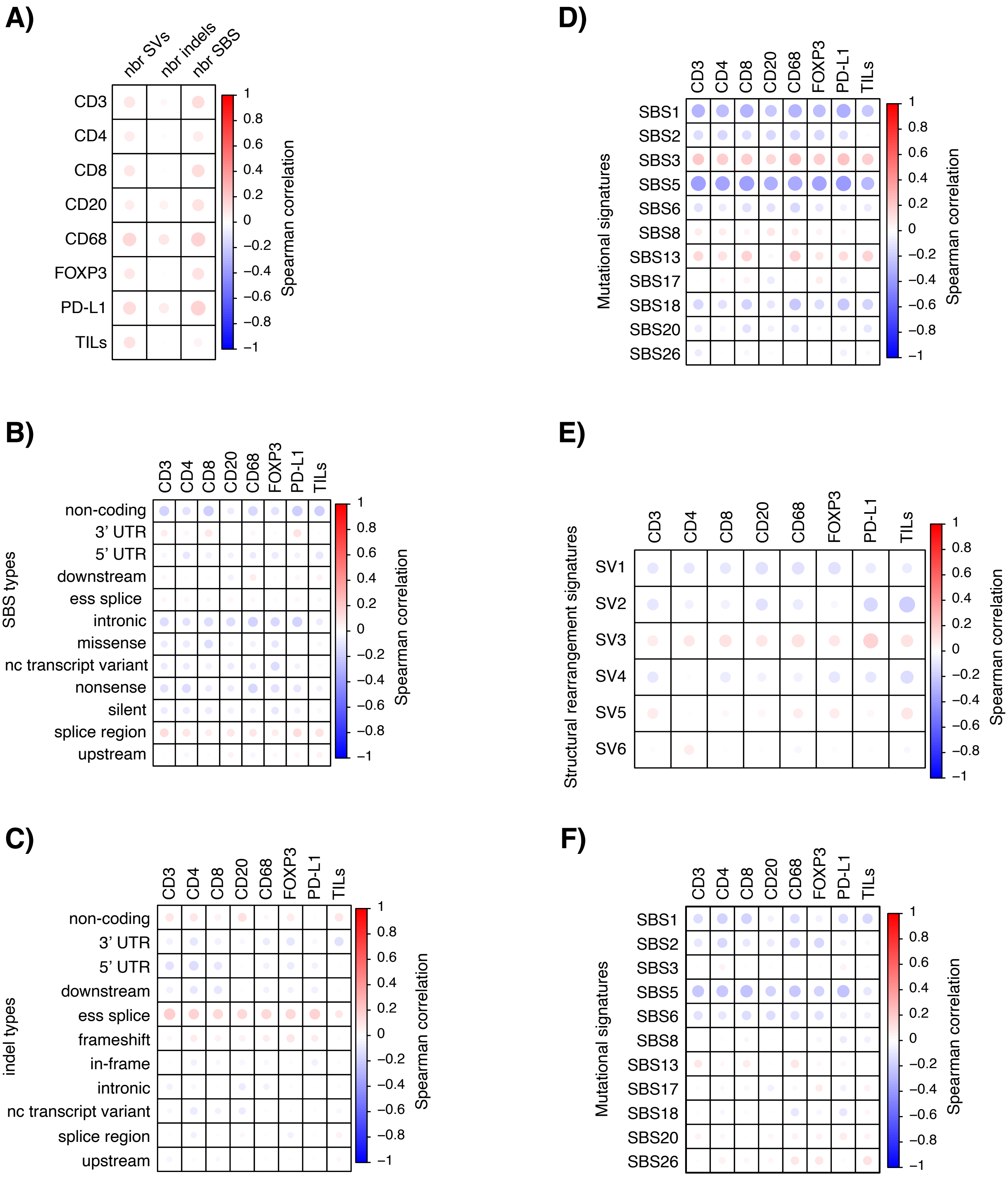

Supplementary Figure 5. Association of TMArQ immune marker cell counts with genetic alterations and mutational signatures in TNBC. Spearman correlation of immune cell counts and TILs versus **A)** total number of single base pair substitutions (SBSs), indels, and structural rearrangements (SVs) in all TNBCs, **B)** proportions of SBS types (number of a type divided by total number of SBSs) in all TNBCs, **C)** proportions of indel types (number of a type divided by total number of indels) in all TNBCs, **D)** SBS mutational signature exposures in all TNBCs, **E)** structural rearrangement signature (SVs) exposures in all TNBCs, and **F)** SBS signature exposures in HRD-positive TNBCs only.
